# Ion Distribution and Cation Exchange at Mica–Electrolyte Interfaces Probed with Deep Potential Molecular Dynamics

**DOI:** 10.1021/acs.chemmater.6c00214

**Published:** 2026-05-04

**Authors:** Sanghyun J. Park, Abhinav S. Raman, Annabella Selloni

**Affiliations:** † Department of Chemistry, 6740 Princeton University, Princeton, New Jersey 08544, United States; ‡ Department of Chemical Engineering, Indian Institute of Technology Madras, Chennai 600036, India

## Abstract

An accurate description of the ion distribution and dynamics at mineral-aqueous solution interfaces is crucial to understanding a variety of technological and environmental phenomena. To obtain better insight, many studies have combined experimental measurements with classical molecular dynamics simulations, yet their interpretation remains limited by the empirical nature of the classical force fields. Here, we investigate the Stern layer structure and cation exchange mechanism at muscovite mica−electrolyte interfaces using nanosecond time scale molecular dynamics simulations based on deep neural network interatomic potentials trained on density functional theory (DFT) data. Focusing on mica with exposed surface K^+^ interfaced with aqueous NaCl and mica with surface Na^+^ interfaced with KCl solution, we find that K^+^ remains predominantly in inner-sphere configurations, while Na^+^ exhibits notable populations in outer-sphere states. Most importantly, our simulations show that contact with an electrolyte solution results in the coadsorption of multiple cation species, making the mica surface locally overcharged and thus reshaping the cation speciation in a manner that enhances the tendency of neighboring surface cations to desorb. These findings are consistent with recent experimental observations that coadsorption of different cation species induces changes in cation speciation and slow kinetics of cation exchange at the muscovite–water interface, providing a basis for their detailed understanding.

## Graphical abstract

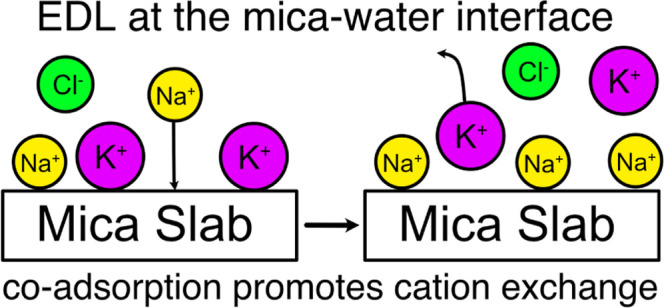

## Introduction

The electrical double layer (EDL) at mineral−aqueous solution interfaces has garnered a great deal of attention, as it plays a major role in many different phenomena, ranging from CO_2_ capture, heterogeneous nucleation, and mineral dissolution to heterogeneous catalysis.^1–9^ Muscovite mica (KAl_2_(Si_3_Al)­O_10_(OH)_2_), a phyllosilicate that can be easily cleaved along the (001) plane, exposing atomically flat surfaces with naturally occurring adsorbed K^+^ to compensate for the negative surface charge,^10,11^ has long been a prototypical system for studies of the EDL at mineral interfaces.^12^ To realize specific surface functionalities for different applications, these K^+^ cations are often exchanged by interfacing the surface with an electrolyte solution, a process that has been the subject of numerous investigations.^13–18^ Although numerous past studies have examined how surface-adsorbed cations modulate the water structure up to 10 Å above the surface,^19–26^ various questions concerning the ion distribution within the EDL and the ion-exchange mechanism have remained unanswered.

According to classical mean-field descriptions, such as the Gouy–Chapman–Stern model, the EDL is divided into two regions: a Stern (or Helmholtz) layer where counterions occupy the innermost region next to the surface, and a diffuse layer with ions located farther away and screening the uncompensated surface charges.^27,28^ While the diffuse layer is relatively well described by classical theories, these models fail to provide a faithful description of the microscopic structure of the Stern layer, as they treat the solvent as a uniform dielectric continuum and neglect the discrete size, hydration structure, and site-specific interactions of ions.^29–33^ In fact, these properties turn out to be decisive factors in determining the intrinsic ion distribution within the Stern layer near mineral–water interfaces. To resolve such atomistic details, many studies have used X-ray reflectivity (XRR) to probe the interfacial structure and determine the electron density profile as a function of the distance from the mica surface.^19,34–38^ Because in many cases such profiles cannot unambiguously distinguish between ions and water, these experiments are often combined with classical molecular dynamics (MD) simulations to better identify how ions within the EDL are distributed and positioned relative to the surface.^26,34,39–41^

Both experiments and simulations have shown that surface cations in the Stern layer can form either an inner-sphere (IS) surface complex, in which cations are directly adsorbed on the surface and partially solvated by water, or an outer-sphere (OS) surface complex, in which cations are fully solvated by water while positioned close to the surface (see Figure 2c).^34,40^ General trends in the preference for IS or OS complexes can be interpreted within the framework of the Hofmeister series, which often provides a useful qualitative basis for understanding competitive ion pairing.^42–44^ In this context, larger monovalent cations such as K^+^ and Rb^+^ are referred to as structure breakers (chaotropes) due to their tendency to disrupt adjacent hydrogen-bond networks, while smaller, more strongly hydrated cations such as Na^+^ and Li^+^ act as structure makers (kosmotropes) by stabilizing hydrogen-bond structures. The preference for IS or OS complexes can be understood as a balance between cation solvation and surface adsorption: kosmotropes favor water solvation because of their more negative hydration free energies and thus exhibit appreciable OS populations, whereas chaotropes, with weaker water solvation, tend to remain primarily in IS configurations. However, the exact speciation and ratio of IS and OS species for kosmotropic ions remain unclear. Indeed, since Na^+^ has the same number of electrons as water and Li^+^ has even fewer, it is challenging to distinguish their signals from those of water in XRR electron density profiles. Moreover, previous classical MD studies have shown that the predicted cation speciation at the muscovite–water interface is sensitive to the chosen classical force field, further complicating efforts to determine the precise structure of the Stern layer.^34,45,46^

Similarly, the cation-exchange mechanism that occurs when mica is interfaced with electrolyte solutions has not yet been fully elucidated. Recent experimental measurements of the exchange between Na^+^ and Rb^+^ revealed that the process occurs over tens of seconds,^16^ implying that real-time cation exchange cannot be captured within the time scale accessible to atomistic MD simulations. These experiments also showed that the desorption rate constant of Na^+^ in RbCl solution is *a* factor of 3 greater than that of Rb^+^ in NaCl solution, indicating that the exchange dynamics may depend on the cation species.^16^ Other experimental studies reported coadsorption of multiple cation species, such as when mica with surface K^+^ ions (K-mica) is interfaced with CsNO_3_ solution, and when the speciation of Sr^2+^ changes due to coadsorption with Na^+^ or K^+^.^17,18^ Altogether, these observations seem to suggest that cation exchange at mica interfaces likely proceeds through coadsorption followed by slow desorption events occurring on experimentally relevant time scales, but the detailed mechanism remains unclear.

In this work, we aim to address the two fundamental questions introduced above: the structure of the Stern layer and the mechanism of cation exchange at the mica–water interface. With the assistance of deep neural network (DNN) interatomic potentials trained on DFT data, we performed nanosecond-time scale MD simulations with ab initio accuracy, allowing us to probe Stern-layer structures and ion dynamics far beyond the reach of conventional ab initio MD and without relying on empirical fitting. Specifically, we investigated two mica systems: K-mica interfaced with pure water and aqueous NaCl and Na-mica (with K^+^ replaced by Na^+^) interfaced with water and KCl solution. This allowed us to resolve the Stern-layer structure in both systems and to observe how adsorbed cations respond when exposed to solutions containing different cations. Our results show that K^+^ cations predominantly form IS complexes, whereas Na^+^ gives rise to a significant OS population. Upon introduction of an electrolyte solution, cations initially in solution tend to coadsorb with those on the surface, driving the surface into an overcharged state. This modifies the cation speciation in a way that enhances the population of the OS and desorbed states, ultimately favoring desorption of all adsorbed cations.

## Results and Discussion

The model systems investigated in this work are shown in Figure 1a,b: K-mica slabs (001) that expose native K^+^ ions and Na-mica slabs in which K^+^ ions are replaced with Na^+^. These surfaces have many possible configurations that can be broadly classified based on the surface cations being arranged either along a row or in a zigzag pattern and occupying ditrigonal cavities including 1 or 2 Al^3+^ arranged in para or meta positions.^47–49^ Here we focus on the two most stable para-Al and meta-Al arrangements where all K^+^ ions initially reside in the more favorable 2-Al cavities comprising two Al atoms and four Si atoms (Figure 1c,d). For our simulations, we use DNN potentials generated according to the Deep Potential (DP) scheme^50,51^ and trained on DFT calculations with the *meta*-GGA SCAN functional, which has been reported to show good accuracy for water and various materials.^52–54^ For production simulations, we use the same (periodic) systems used to train the DPs, which consist of a single layer of mica (001) 2 × 2 with 4 K^+^ or 4 Na^+^ exposed on each surface to achieve a net charge of zero. The adequacy of a single layer slab model to describe the mica surface was verified in previous studies.^11,48^ The mica slabs are interfaced with 16 Å thick slabs of NaCl or KCl aqueous solution at concentrations of 0.0 and 1.5 M, corresponding to zero and three ion pairs per periodic cell, respectively, as in the representative snapshot shown in Figure 1e. We intentionally kept the system size identical to that of the training set to faithfully capture long-range electrostatic interactions while retaining the original short-range DP architecture with a descriptor cutoff radius set to 6 Å.^50^

**Figure 1:**
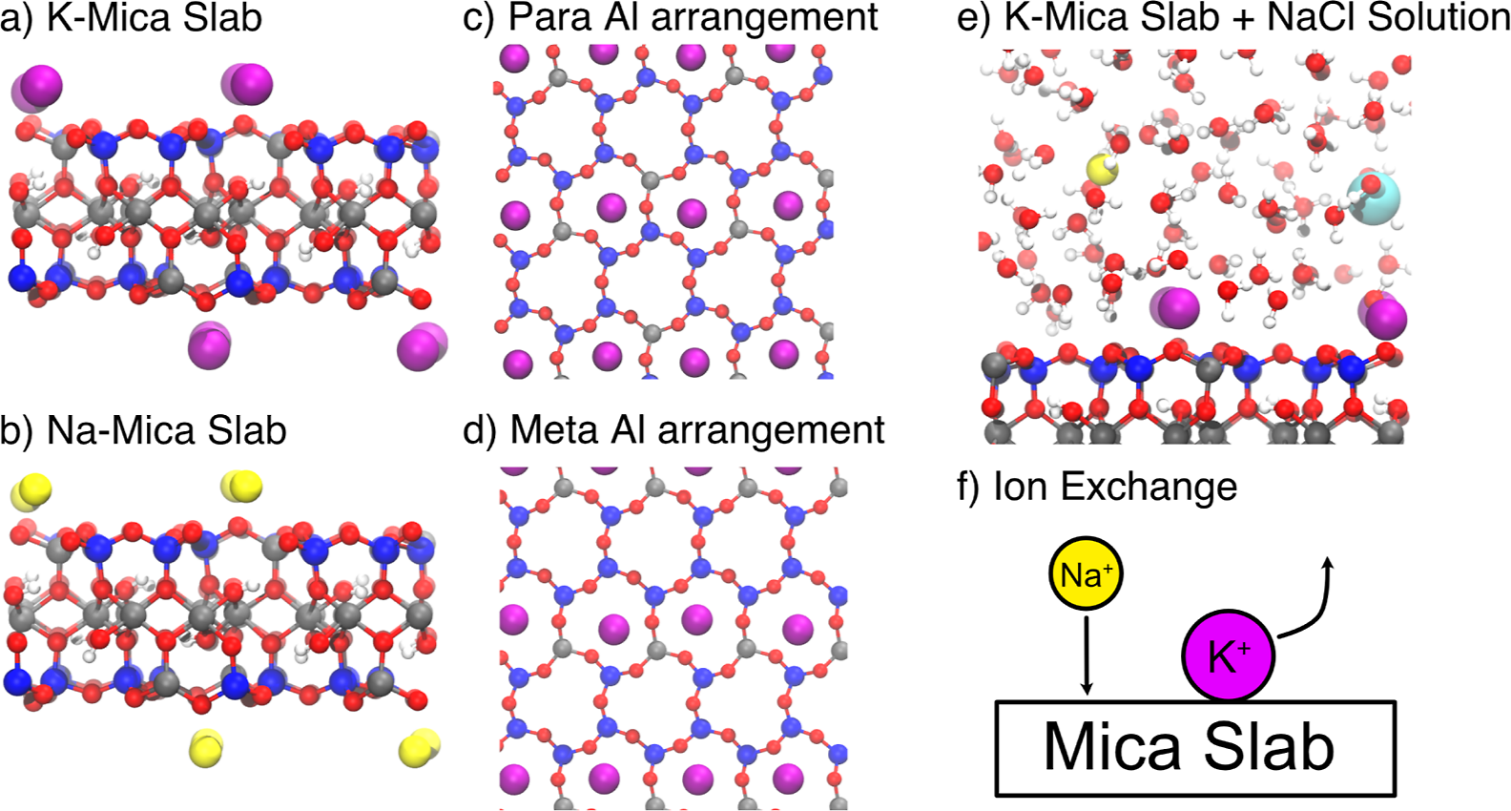
Snapshots of single-layer (001) K-mica (a) and Na-mica (b) slabs used in the production simulations. On each surface, 4 K^+^ (purple) or 4 Na^+^ (yellow) are initially adsorbed. Distribution of Al atoms (gray) for: (c) the para-Al structure, where Al atoms occupy opposite sites of the same cavity, and (d) the meta-Al structure, where Al atoms are in meta positions, separated by one Si atom (blue). All K^+^ or Na^+^ ions are initially positioned in the ditrigonal cavities with 2 Al atoms (2-Al) which are energetically more favorable than those containing only one Al.^47,48^ (e) Representative snapshot of a K-mica slab interfaced with aqueous 0.5 M NaCl solution (corresponding to 1 NaCl pair per unit cell). (f) Schematic illustration of the real-time cation exchange mechanism in which surface adsorbed K^+^ is replaced by Na^+^.

We also note that the 16 Å electrolyte slabs can be divided into three regions based on the distance from the mica surface: 0–3 Å for the Inner-Sphere (IS) region, 3–6 Å for the Outer-Sphere (OS) region, and distances beyond 6 Å for the diffuse region (see Figure 2). The biggest limitation arises from the rather thin diffuse region, which consists of only 4 Å of the electrolyte slab and may undergo large local concentration fluctuations whenever a cation desorbs from the IS or OS states. Such an effect is expected to bias the system toward readsorption and make rigorous sampling of the fully equilibrated speciation difficult. As a result, the IS and OS populations may be somewhat overestimated relative to the desorbed population. Nevertheless, the overall thickness is sufficient to resolve the IS and OS speciation of K^+^ and Na^+^ within the Stern layer and to quantify the free energy of cation migration from the IS to OS or desorbed states (see below). Thus, we expect the mechanistic implications of this work to remain robust, since the largest barrier to cation desorption is associated with escape from tightly bound IS species.

**Figure 2:**
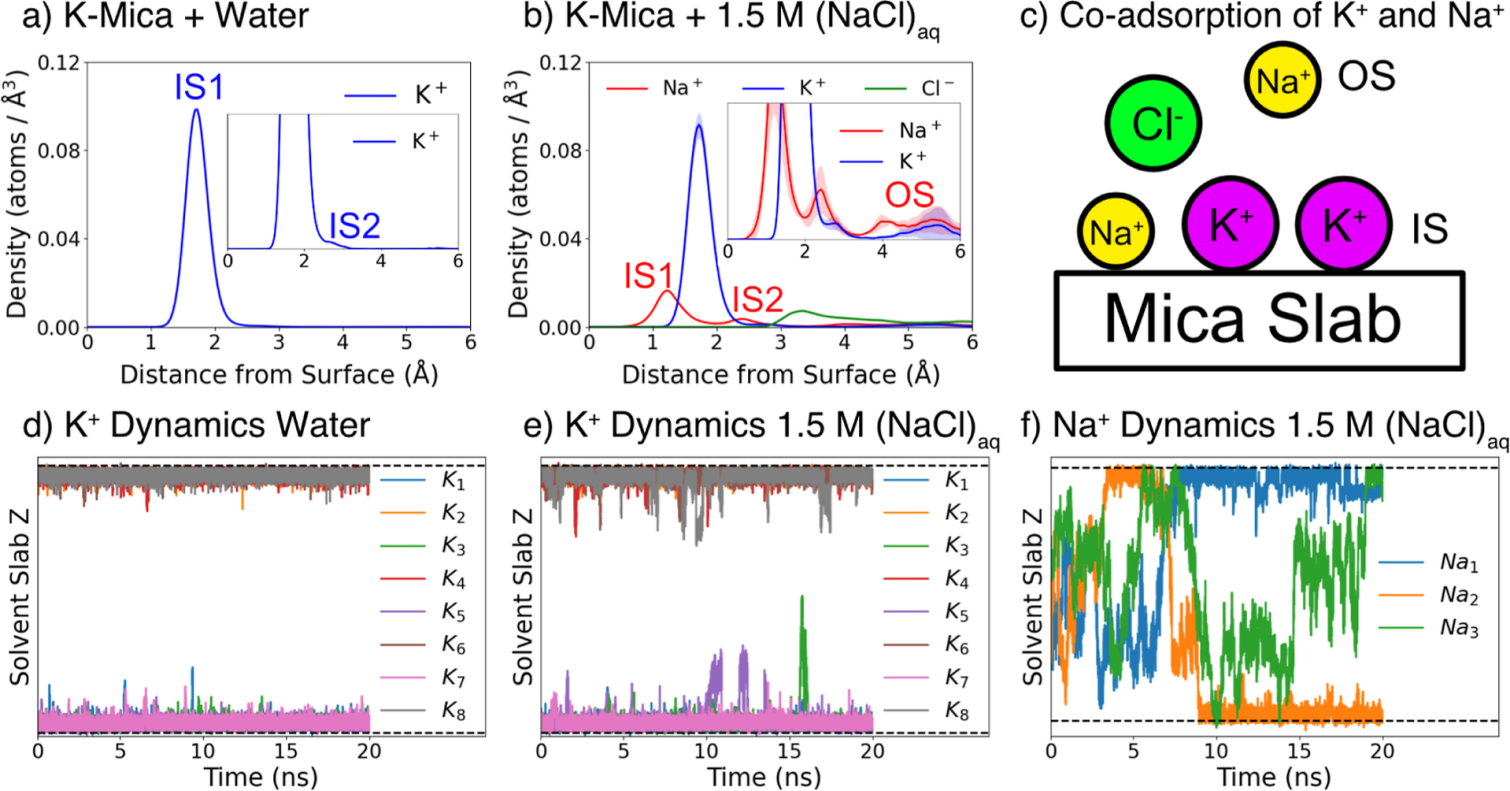
(a,b) Ion density profiles as a function of distance from the mica surface for K-mica interfaced with pure water (a) and 1.5 M aqueous NaCl (b). The inner-sphere IS1, IS2, and outer-sphere OS peaks are labeled at their respective positions. While chaotropic K^+^ exhibits a dominant IS1 peak, kosmotropic Na^+^ displays noticeable IS2 and OS populations. (c) Illustration of coadsorption of both the original K^+^ and the added Na^+^ cations on the mica surface. IS surface complexes are directly adsorbed and partially solvated by water, whereas OS complexes are fully solvated. (d–f) *z*-axis positional dynamics of K^+^ and Na^+^ ions in K-mica systems. Although K^+^ generally remains strongly adsorbed, the introduction of 1.5 M NaCl_aq_ induces occasional desorption and larger fluctuations, while Na^+^ exhibits pronounced positional fluctuations across different adsorption states. The two horizontal dashed lines near the top and bottom of each panel represent the two surfaces of the mica slab.

Additional information on the training and validation of our DPs and the DPMD simulations is provided in Materials and Methods and the Supporting Information, while a more detailed discussion of the importance of including explicit long-range interactions at heterogeneous solid–electrolyte interfaces is reported in ref 55. On the basis of our results, the overall trends for para-Al and meta-Al arrangements were nearly identical; hence, only the results for the most stable para-Al arrangement are presented in this paper. Results for the meta-Al arrangement are provided in the Supporting Information.

### K-Mica Interfaced with Water and Aqueous NaCl Solution

Two types of IS complex are typically observed in the Stern layer of the mica–water interface: IS1, with cations located in the center of the ditrigonal cavities, and IS2, with cations located directly above the Al sites and toward the edge of the cavities.^34^ While IS1 is commonly observed for all cations, the occurrence of IS2 strongly depends on the cation species. To elucidate the preferred speciation of K^+^, we calculated the density of K^+^ as a function of the distance from the surface for K-mica interfaced with pure water (Figure 2a). The dynamics of K^+^ for the same system are shown in Figure 2d. As seen here, K^+^ ions rarely desorb from the surface and form a highly stable IS1 species throughout the 20 ns DPMD simulation. K^+^ predominantly favors IS1 over both IS2 and OS, which are rarely observed, a behavior consistent with the fact that K^+^ is relatively structure breaking (chaotropic) and therefore favors surface adsorption over water solvation.

Figure 2b shows the distributions of K^+^, Na^+^, and Cl^–^ ions when the K-mica surface is interfaced with a 1.5 M NaCl solution (corresponding to 3 NaCl pairs per unit cell). According to these density profiles, the added Na^+^ exhibits a significant population of IS2 and OS species, highlighting its stronger tendency toward water solvation. This result is in good agreement with previous classical MD studies of Stern layers at mica interfaces.^34,40^ This is also in accordance with the Hofmeister series: Na^+^ is more structure making (kosmotropic) than K^+^, thus more strongly favoring water solvation. Interestingly, K^+^ is subtly affected by the presence of external ions, with a small amount of IS2 and OS species emerging in the density profile. To provide a more rigorous quantification of the speciation, we calculated the average fraction of both K^+^ and Na^+^ in the different adsorption states, as shown in Table 1. As expected, Na^+^ shows a much larger population in the OS and desorbed states, and the addition of NaCl slightly decreases the fraction of K^+^ in the IS state.

**Table 1:** Average Fractions of K^+^ or Na^+^ Ions in the Different Adsorption States from Metadynamics (First Column) and DPMD (all Other Columns) in K-Mica and Na-Mica Systems, and Their Corresponding Surface Coverages

system	IS (metadyn.)	IS (DPMD)	OS (DPMD)	desorbed (DPMD)	coverage (DPMD)
K^+^ in K-mica water	100%	97.3%	0.2%	2.5%	0.975
K^+^ in K-mica 1.5 M (NaCl)_aq_	98.3%	93.5%	3.2%	3.3%	0.967
Na^+^ in K-mica 1.5 M (NaCl)_aq_	74.8%	68.9%	18.0%	13.1%	0.326
Na^+^ in Na-mica water	68.9%	76.9%	11.1%	12.0%	0.880
Na^+^ in Na-mica 1.5 M (KCl)_aq_	58.4%	59.5%	17.3%	23.1%	0.768
K^+^ in Na-mica 1.5 M (KCl)_aq_	99.9%	96.0%	0.9%	3.0%	0.363

These observations are well reflected in the time evolution of the *z*-coordinates of the K^+^ and Na^+^ ions, representing their distances from the surface, shown in Figure 2d–f. We can see that the K^+^ cations become more susceptible to transient desorption when the surface is in contact with a NaCl solution but still remain strongly adsorbed on the surface. In contrast, the added Na^+^ ions are much more mobile, often transitioning between IS, OS, and desorbed species due to their kosmotropic character. Most importantly, we observed coadsorption of K^+^ and Na^+^, with both cations occupying surface cavity sites simultaneously. To quantify this finding, we calculated the cation coverage of the mica surface, which is shown in the last column of Table 1. The coverage of K-mica interfaced with water stays close to one since K^+^ seldom migrate out of the IS state. When interfaced with a 1.5 M NaCl solution, however, the total cation coverage increases to ∼1.3 due to coadsorption, signifying surface overcharging. This phenomenon arises because the mica surface contains more adsorption sites than are initially occupied by native K^+^ ions, although the coverage may be somewhat overestimated due to the small solution slab size.

Given the very long time scale associated with ion dynamics often observed in experiments,^16^ 20 ns of unbiased DPMD may be insufficient to obtain fully converged statistics. Therefore, we performed enhanced sampling simulations to predict the free energy profile of cation diffusion and desorption on the mica surface. Specifically, we carried out well-tempered metadynamics simulations using two collective variables (CVs): the K^+^–Al coordination number (CN) to monitor K^+^ diffusion between 2-Al and 1-Al cavities, and a CV designed to track the numbers of K^+^ in the IS state. Details of the metadynamics simulations and CV definitions are provided in the Supporting Information. The resulting two-dimensional free energy surfaces for two NaCl concentrations are shown in Figure 3a,b, where darker blue colors indicate lower free energy states. In the absence of Na^+^ and Cl^–^, surface K^+^ ions frequently relocate from more favorable 2-Al cavities to less favorable 1-Al cavities, yielding an average K^+^–Al CN of ∼12, in good agreement with previous results from our group^48^ (note that our simulation cell contains 8 K^+^ ions, so K^+^–Al CN = 16 when all K^+^ are in 2-Al cavities.) When K-mica is interfaced with 1.5 M NaCl solution, the average K^+^–Al CN decreases to ∼10 due to the presence of coadsorbed Na^+^ ions competing for the more favorable 2-Al cavity sites. At the same time, migration of K^+^ to the OS and desorbed states is promoted in 1.5 M NaCl_aq_, as evidenced by the lower free energies associated with states that exhibit fewer K^+^ ions in the IS state.

**Figure 3:**
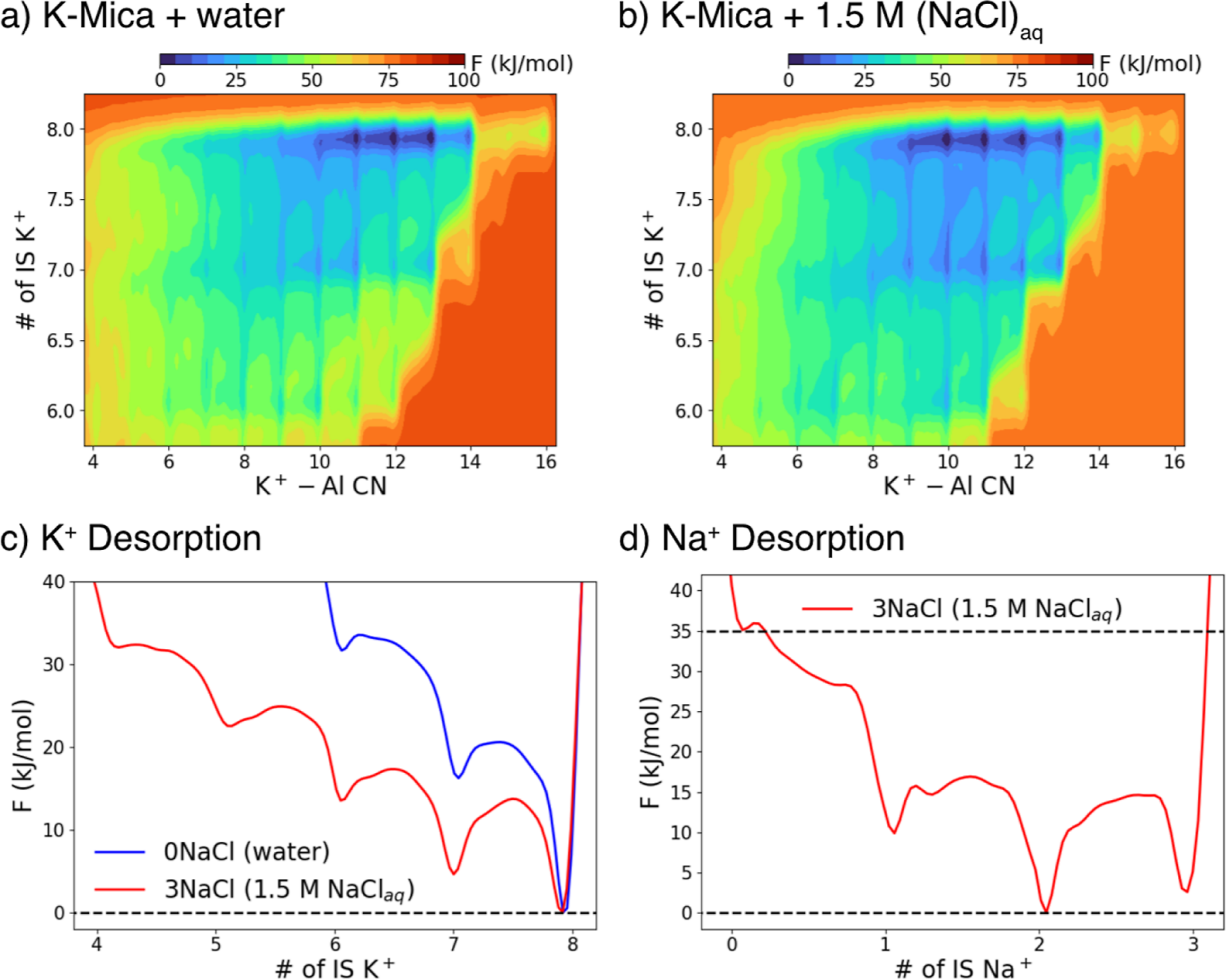
Two-dimensional free energy profiles projected onto two CVs representing the number of IS K^+^ ions and the K^+^–Al CN in K-mica interfaced with either pure water (a) or aqueous 1.5 M NaCl (b). Note that our simulation cell contains 8 K^+^ ions, so that K^+^–Al CN = 16 when all K^+^ are in 2-Al cavities. Darker blue hues correspond to lower free energy states. In (b), the presence of 1.5 M NaCl causes K^+^ to more frequently occupy 1-Al cavity sites, reflected by a decrease in the preferred K^+^–Al CN, and also increases the propensity of K^+^ for desorption. (c,d) One-dimensional free energy profiles projected onto CVs describing the number of IS K^+^ or Na^+^. Although K^+^ predominantly favors full IS adsorption, the presence of 1.5 M NaCl lowers the free energy associated with K^+^ desorption. In contrast, the free energy profile of Na^+^ indicates pronounced fluctuations between the IS, OS, and desorbed states.

To substantiate this behavior, we constructed a one-dimensional free energy profile along the CV that tracks K^+^ IS adsorption (Figure 3c). This process was done by performing a Boltzmann-weighted integration over one of the CVs. The resulting profile shows a clear decrease in the free energy of K^+^ escape from IS when pure water is replaced by a 1.5 M NaCl_aq_ solution, although the configuration with all K^+^ in the IS state remains the most stable. The free energy profile obtained from an analogous metadynamics simulation for the added Na^+^ is shown in Figure 3d. The most favorable state features two of the three Na^+^ cations in the IS configuration, reflecting its more kosmotropic nature. The IS adsorption fractions obtained by the Boltzmann averaging of the metadynamics free energy profiles and by direct averaging of the DPMD trajectories are summarized in Table 1. Overall, these results clearly demonstrate that coadsorption and surface overcharging make adsorbed K^+^ increasingly susceptible to desorption.

### Na-Mica Interfaced with Water and Aqueous KCl Solution

The Na^+^ density profile for Na-mica interfaced with pure water is shown in Figure 4a. Unlike K^+^ in K-mica, which essentially forms a single IS1 peak, Na^+^ displays appreciable populations of both IS2 and OS states. In addition, IS Na^+^ exhibits pronounced in-plane mobility similar to K^+^, resulting in frequent hopping between 1-Al and 2-Al ditrigonal cavities. When Na-mica is interfaced with 1.5 M KCl_aq_ (corresponding to 3 KCl pairs per unit cell), the density profiles (Figure 4b) show that the added K^+^ ions become stably adsorbed, as evidenced by the single sharp IS1 peak. In contrast, the IS1 peak of Na^+^ decreases markedly while the IS2 and OS peaks increase, which is also supported by the significant increase in the OS and desorbed fractions shown in Table 1. These results indicate that as K^+^ occupies ditrigonal cavity sites on the surface, Na^+^ in IS states becomes increasingly prone to desorption, thus notably increasing its OS and desorbed populations. Co-adsorption induced surface overcharging is also observed in this system, with a total cation coverage of ∼1.131, a value lower than that of the K-mica systems. Although the qualitative behavior mirrors that of K-mica, the desorption tendency is considerably more pronounced in Na-mica.

**Figure 4:**
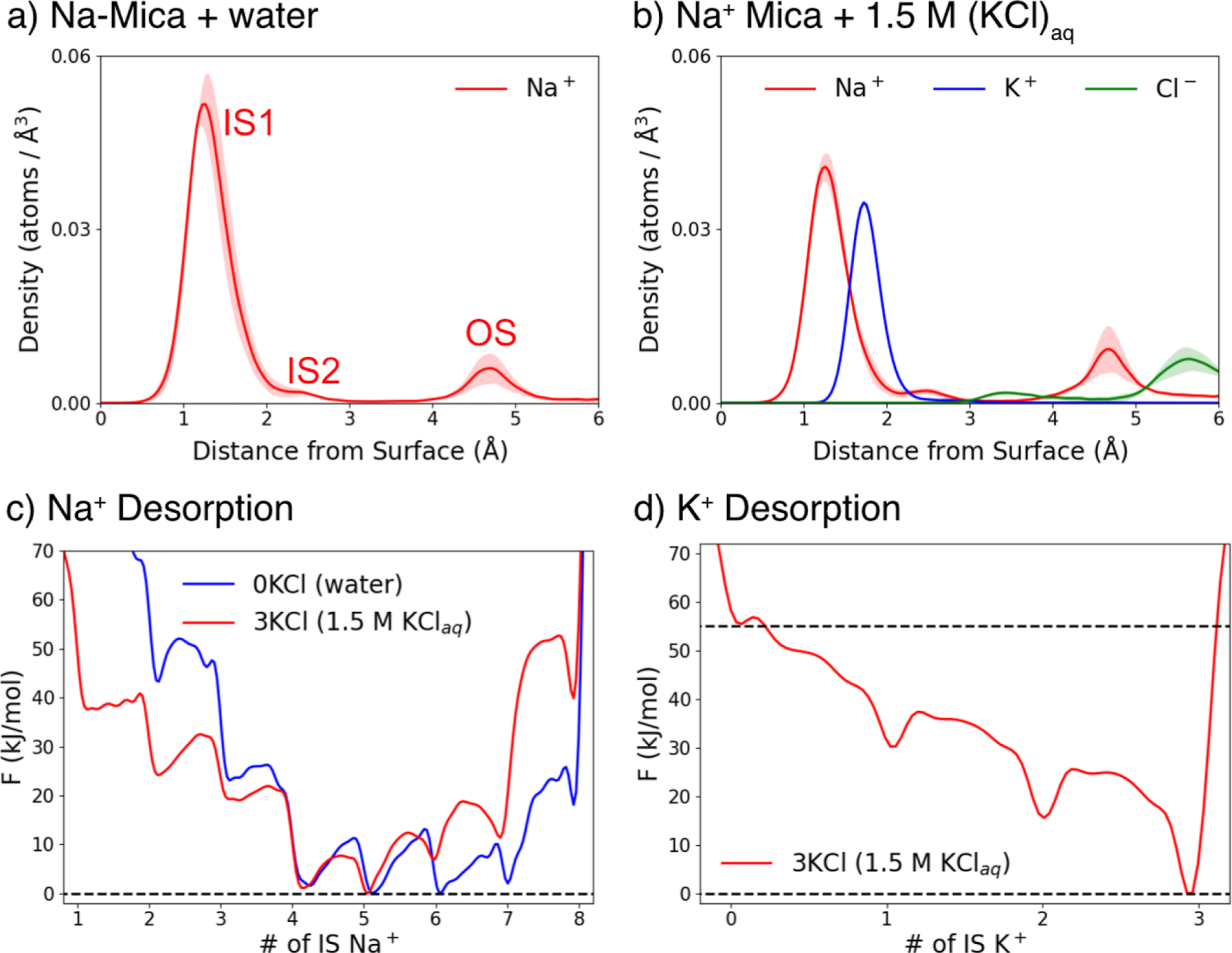
(a,b) Ion density profiles as a function of distance from the mica surface for Na-mica systems interfaced with either pure water or 1.5 M aqueous KCl. Although Na^+^ primarily occupies the IS1 state, it also exhibits appreciable populations in IS2 and OS states. Upon addition of 1.5 M KCl, chaotropic K^+^ remains adsorbed in the IS1 state, while the Na^+^ IS1 population decreases and the IS2 and OS peaks become more pronounced. (c,d) One-dimensional free energy profiles projected onto CVs depicting the number of K^+^ or Na^+^ in the IS configuration. In the absence of KCl, Na^+^ displays prominent dynamics along the surface-normal direction, reflected by small free energy differences among states with 4–7 Na^+^ in the IS state. When the system is interfaced with 1.5 M KCl_aq_, states with fewer IS Na^+^ become more favorable, while K^+^ predominantly favors IS adsorption.

To provide a more robust picture of the enhanced Na^+^ desorption in Na-mica, we performed enhanced sampling simulations using CVs that track the number of IS K^+^ and Na^+^, analogous to those done for K-mica. The resulting one-dimensional free energy profiles are shown in Figure 4c,d. For Na-mica, a substantial fraction of Na^+^ avoids IS occupation and instead favors complete water solvation, as reflected by the most favorable state containing only 5–6 Na^+^ in the IS state. Upon introduction of 1.5 M KCl_aq_, states with more than five IS adsorbed Na^+^ become less favorable, while states with fewer IS adsorption become more favorable, signifying a clear enhancement in Na^+^ desorption. In contrast, the added K^+^ strongly prefers IS adsorption. From these free energy profiles, we calculated Boltzmann averages of the number of IS Na^+^ at different concentrations of KCl_aq_, summarized in Table 1. As expected, a steeper decrease in the average fraction of IS Na^+^ is observed relative to K^+^ in K-mica, indicating a more pronounced effect of the electrolyte solution on Na^+^ desorption, despite the fact that Na^+^ and K^+^ lie close to each other in the Hofmeister series and their solvation free energies differ by only approximately 20%.

### Cation Exchange

According to a recent experimental study of the dynamics of cation exchange in Rb-mica interfaced with NaCl and in Na-mica interfaced with RbCl, the time scale of the exchange process ranges from seconds to tens of seconds.^16^ Furthermore, the kinetic rate constant for the desorption of Rb^+^ was measured to be roughly three times slower than for Na^+^. Although the observed time scale of the exchange process is far too slow to be captured directly by DPMD, the observed difference between the desorption rates of Rb^+^ and Na^+^ is consistent with our observations for Na^+^ and K^+^. Additionally, another recent experimental study reported coadsorption of K^+^ and Cs^+^ after exposure of K-mica to CsNO_3_ solution.^18^ Together, these experimental findings support the idea that the coadsorption and overcharge induced enhancement of desorption observed in our work likely play a key role in the overall mechanism of ion exchange on experimental time scales.

Based on our simulations, when new cations are introduced into the interfacial region, these cations tend to coadsorb on the surface, which becomes overcharged. This is mainly due to the fact that the mica surface possesses more available adsorption sites than those occupied initially by K^+^ or Na^+^ and at the same time the anions in the Stern layer help stabilize the excess surface charge. However, once the surface becomes overcharged, the speciation of all cations shifts in a direction that enhances both the OS and desorbed populations. As a result, adsorbed cations become more susceptible to desorption, making it reasonable to expect that under typical cation exchange conditions, the original cations will gradually be replaced by the incoming species (Figure 5). Furthermore, our simulations show that the enhancement of desorption is more pronounced for kosmotropic ions than for chaotropic ions. Consequently, replacing the kosmotropic Na^+^ with the chaotropic Rb^+^ is more favorable than the reverse substitution, consistent with the experimentally observed larger Na^+^ desorption rate constant.^16^ This is also consistent with the recent experiment showing a larger shift in the speciation of Sr^2+^ when the more chaotropic Rb^+^ is added compared to Na^+^.^17^ We expect these conclusions to remain valid despite the inherent limitations of our simulations, thus providing meaningful insights into cation speciation and ion exchange mechanisms at the muscovite–water interface.

**Figure 5:**
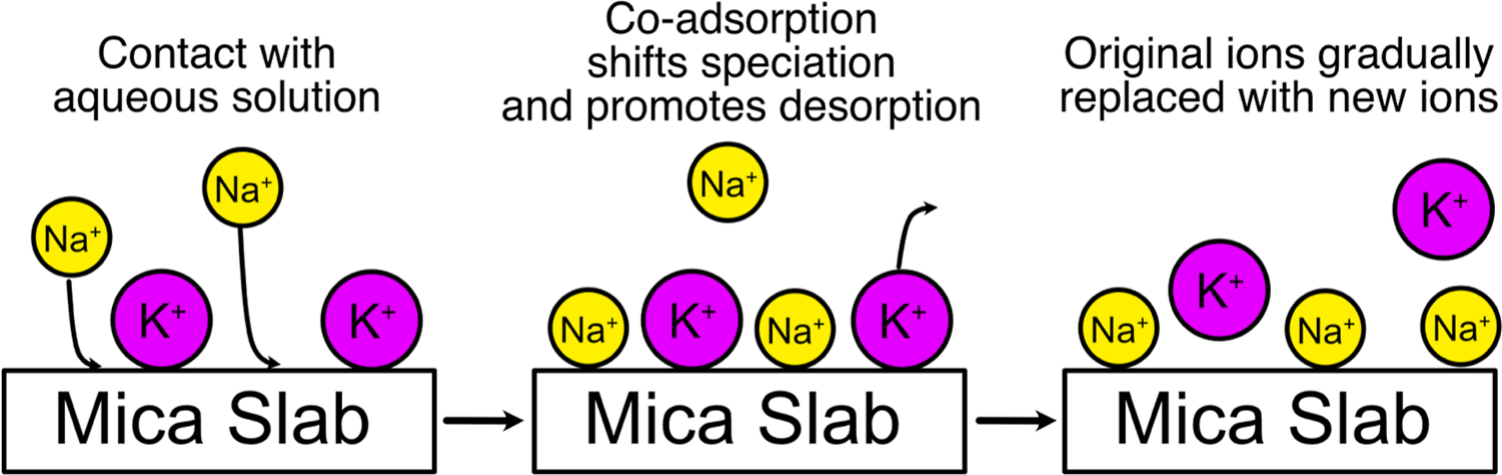
Schematic illustration of the proposed mechanism for ion exchange dynamics at mica–electrolyte interfaces. When mica is exposed to electrolytes, incoming cations become coadsorbed on the surface, leading to surface overcharging. This reshapes the speciation of all adsorbed cations in a direction that increases the susceptibility to desorption, and on experimental time scales, the original surface species are gradually replaced by the incoming ions.

## Conclusion

In summary, we have examined the structure of the Stern layer and the associated ion dynamics of both K-mica and Na-mica in contact with pure water and aqueous electrolyte solutions. Our results reveal that K^+^ strongly favors the IS1 configuration, whereas Na^+^ exhibits significant populations of both OS and desorbed states. This difference well reflects the expected Hofmeister trend, in which kosmotropic Na^+^ favors water solvation more strongly than chaotropic K^+^, leading to a greater propensity for OS states and desorption. However, IS still remains the dominant species for Na^+^, with the sum of OS and desorbed fractions ranging from 20–40% depending on the salt concentrations. More importantly, interfacing mica with an aqueous electrolyte solution leads to pronounced coadsorption of multiple cation species, driving the mica surface into an overcharged state. Surface overcharging, in turn, shifts the balance between IS and OS populations in *a* direction that significantly enhances cation desorption, with the magnitude of this effect depending on the Hofmeister trend of the cations. Taken altogether, these observations motivate a cation-exchange mechanism in which the initial native cations are gradually replaced on experimental time scales through cycles of coadsorption, overcharging, and facilitated desorption. Overall, the proposed mechanistic picture aligns well with experimentally reported coadsorption behavior and relative desorption rates for kosmotropic/chaotropic ions, providing insights into real-time ion exchange on the mica surface.

## Materials and Methods

Our DPs^50,51^ were generated using an iterative active learning scheme in which configurations were sampled on the fly using DP-based molecular dynamics (DPMD) at different temperatures.^56^ During the exploration phase, configurations that showed large deviations in forces between three DPs initialized with different random seeds were selected for labeling and added to the training set. The active learning cycle was terminated when the mean force deviation fell below 0.05 eV Å^–1^ over a 100 ps DPMD run at 300 K. We further used enhanced sampling to enlarge the training set by thoroughly probing the ion-dynamics configuration space. Throughout both training and production simulations, the force deviations between the multiple DPs were monitored to assess whether the models were entering regions of higher uncertainty that may require additional training. For the labeling step, we employed DFT calculations with the *meta*-GGA SCAN functional.^52–54^ These calculations were performed using the plane wave-pseudopotential scheme as implemented in the Quantum Espresso package,^57^ with ONCV norm-conserving pseudopotentials and a 110 Ry cutoff for the wavefunctions.^58^

The initial data set used to initiate the active learning procedure was adapted from previous studies by our group, including configurations of bulk water, bulk mica and mica–water interfaces for different Al and K^+^ arrangements.^48,49^ For active learning, we chose to explore two of the most stable Al arrangements reported in these studies, namely the para-Al and meta-Al arrangements, where all K^+^ cations occupy the most favorable ditrigonal 2-Al cavities (see Figure 1c,d). The configurations used in active learning comprised a single layer of (001) 2 × 2 mica slabs with 4 K^+^ or 4 Na^+^ cations exposed on each side (Figure 1a,b), interfaced with a 16 Å thick slab of 0.5 or 1.5 M electrolyte solution. For the mica slab with surface K^+^ (K-mica), the electrolyte was a NaCl solution, while the Na-mica slab was interfaced with KCl solution. After the active learning procedure was complete, we also performed enhanced sampling to collect configurations with different numbers of adsorbed K^+^ and Na^+^ cations and incorporated them into the training set. Such a meticulous training procedure ensured satisfactory sampling of the relevant configuration space for exploring the ion-exchange dynamics at mica-electrolyte interfaces. The final data set consisted of approximately 50,000 configurations, with further details provided in the Supporting Information.

Our training set energy and force RMSEs were approximately 0.8 meV atoms^–1^ and 0.13 eV Å^–1^, respectively, very similar to the values obtained in previous studies.^48^ To validate the finalized DPs, we built a data set by sampling configurations from 20 ns unbiased DPMD trajectories and compared the energy and force predictions of ab initio SCAN with those of our DPs. Both the validation set energy and force errors were smaller than the corresponding training set errors, indicating that our DPs are capable of predicting energies and forces for relevant configurations with high fidelity. Furthermore, we compared the radial distribution functions of the aqueous KCl solution and K-mica interfaced with NaCl solution predicted by AIMD-SCAN and DPMD and found that they are in excellent agreement. All of these validation tests suggest that the trained DPs are capable of reproducing SCAN-level accuracy for mica−electrolyte solution interface systems.

DPMD simulations with the trained DPs were performed using LAMMPS interfaced with the DeepMD-kit package.^59^ We used the same mica−electrolyte interfacial systems as those used during the training to ensure the accurate treatment of long-range electrostatic interactions, which are crucial to accurately describe the structures of the Stern layer at heterogeneous interfaces.^55,60^ All simulations were carried out in the *NVT* ensemble with the Nosé-Hoover chain thermostat that maintained the temperature at 300 K.^61^ All H_2_O molecules were replaced with D_2_O to mitigate integration errors when using a time step of 0.5 fs. DPMD trajectories of 20 ns were generated using three independently trained DPs, with all reported equilibrium properties calculated between 5 and 20 ns and averaged across the three models. Additionally, enhanced sampling calculations were performed using one of the three DPs and the PLUMED package interfaced with LAMMPS.^62^ A detailed description of the production simulations, including unbiased DPMD and enhanced sampling, is provided in the Supporting Information.

## Supplementary Material



## Data Availability

The following Github repository contains all the DPs used in this work, alongside the training set, validation set, and input files to train a DP potential. In addition, input files for the Quantum ESPRESSO, LAMMPS, and PLUMED program suites are included for reference. All data are available at: https://github.com/sanghyunjonathan/mica-electrolyte-interface.git

## Notes

The Supporting Information contains the parameters used in the simulation, additional details of the simulations methodology and Supporting Information analysis (PDF)
