# Supplementary material for: Ion Distribution and Cation Exchange at Mica–Electrolyte Interfaces Probed with Deep Potential Molecular Dynamics
Source: Chem Mater. 2026 May 4;38(10):5049–57. doi: 10.1021/acs.chemmater.6c00214 (PMC13217552; doi:10.1021/acs.chemmater.6c00214)
Supplement: Supplementary file 1 [file cm6c00214_si_001.pdf]

Supporting Information for "Ion Distribution and Cation Exchange at Mica–Electrolyte Interfaces Probed with Deep Potential Molecular Dynamics"

Sanghyun J. Park,† Abhinav S. Raman,‡ and Annabella Selloni*,†

†*Department of Chemistry, Princeton University, Princeton, NJ, 08544, USA*

‡*Department of Chemical Engineering, Indian Institute of Technology Madras, Chennai, 600036, India*

E-mail: aselloni@princeton.edu

Table of Contents

1 Methods and Models **3**

1.1 DFT Calculations 3

1.2 Training and Active Learning 3

1.3 Enhanced Sampling 4

2 DP Error Analysis and Validation **8**

2.1 Training and Validation Set Errors 8

2.2 Validations on Structures 8

3 Additional Analysis of the Equilibrium Trajectories **12**

4 Additional Analysis of the Enhanced Sampling Simulations **20**

References **27**

1 Methods and Models

1.1 DFT Calculations

Our general methodology closely follows that of our previous studies of muscovite mica–water interfaces.[1,2] The labeling step was carried out using the SCAN functional implemented in the Quantum Espresso program.[3,4] Core electrons were treated with the ONCV norm-conserving pseudopotentials with a wavefunction cutoff of 110 Ry.[5] A small Gaussian smearing of 0.02 Ry was applied to assist convergence without affecting energies and forces. Γ-point sampling was used for all interfacial systems due to their large system size. The initial configurations for the first training step were taken from CP2K AIMD simulations using the PBE-D3 functional.[6,7] Specifically, a MOLOPT double-zeta basis set and a 500 Ry plane-wave cutoff were employed along with GTH pseudopotentials to treat the core electrons.[8] AIMD trajectories were propagated for 10 ps at 300 K using the Nosé-Hoover chain thermostat.[9]

1.2 Training and Active Learning

We trained an initial coarse DP using configurations taken from AIMD simulations together with configurations adapted from previous studies by our group. This DP was used to initiate an iterative active learning cycle[10] for the following systems: K-mica interfaced with 0.5 M and 1.5 M NaCl solutions and, similarly, Na-mica interfaced with 0.5 M and 1.5 M KCl solutions, where 0.5 and 1.5 M NaCl/KCl correspond to 1 and 3 NaCl/KCl pairs per unit cell, respectively. Both meta-Al and para-Al arrangements on the mica surface were considered with all K^+ ions initially placed on the more favorable 2-Al ditrigonal cavities. The solid slab consists of a single (001) 2×2 mica layer with four K^+ or four Na^+ ions exposed on each side, interfaced with a 16 Å thick slab of electrolyte solution, with concentration 0.0 M, 0.5 M, or 1.5 M and the solution density fixed at 1 g/cm^3. The DP neural network comprises a $25 \times 50 \times 100$ embedding network followed by a $120 \times 120 \times 120$ fitting network.[11] Training was performed for 2,000,000 steps using a batch size of one, while the learning rate was decreased

from 0.005 to 1.75×10^{-7}.

Each active learning step consists of an exploration phase, during which the configurations were sampled by running DPMD simulations at temperatures ranging from 200 to 600 K for up to 100 ps. During this phase, configurations with maximum force deviations between 0.1 and 0.8 eV Å^{-1} were selected. These configurations were then labeled using the SCAN functional implemented in the Quantum Espresso package and added to the training set.[3,4] This iterative scheme was concluded when the mean force deviation fell below 0.05 eV Å^{-1} over 100 ps-long DPMD trajectories.

In addition, an accurate description of the ion desorption and adsorption dynamics using the trained DPs requires a wide range of configurations to be included in the training set. To achieve this, we added configurations from well-tempered metadynamics simulations in which we systematically varied the number of adsorbed K^+ and Na^+ cations using a CV that represented the number of adsorbed cations. The final training set consisted of approximately 50,000 configurations, with details listed in Table S1.

1.3 Enhanced Sampling

All enhanced sampling simulations were performed using only one of the three trained DPs. To estimate the free energy profile of Na^+ desorption in K-mica interfaced with 1 NaCl solution (see Section 4, Additional Analysis of the Enhanced Sampling Simulations, below), we employed well-tempered metadynamics with a CV defined as the z-distance between a selected Na^+ cation and Al atoms located at the center of the slab. Specifically, Na^+ desorption was probed on meta-Al and para-Al K-mica slabs interfaced with 0.5 M NaCl solution (1 NaCl per unit cell). To prevent interaction between the Cl^- anion and Na^+, the anion was restrained to remain close to the opposite surface throughout the simulations. Gaussians with a height of 1.0 kJ mol^{-1} were deposited every 1000 time steps, with a bias factor set to 10 at 300 K.

Similarly, to estimate the free energy profile of the cation exchange between surface

Table S1: Configurations in the training set (* Taken from Ref.[1] and Ref.[12])

System	# of frames
Bulk Water	800*
Bulk Mica	750*
Mica–Vacuum Interface	1229*
$64H_2O$ + 1NaCl	1709*
$64H_2O$ + 2NaCl	1975*
$64H_2O$ + 4NaCl	965*
$64H_2O$ + 6NaCl	1000*
K-mica–Water Interface	3336*
K-mica–1NaCl Solution Interface	15985
K-mica–3NaCl Solution Interface	16463
Na-mica–1KCl Solution Interface	1058
Na-mica–3KCl Solution Interface	1083
$64H_2O$ + 2NaCl + 2KCl solution	2909

adsorbed (IS) K^+ and Na^+ (see Section 4 below), we performed well-tempered metadynamics simulations using two CVs: $z_1 - z_2$ and $z_1 + z_2$, in which z_1 represents the z-distance between a selected K^+ ion and surface O atoms, and z_2 is the z-distance between the Na^+ ion and surface O atoms. We applied a restraint to prevent both K^+ and Na^+ from approaching the opposite surface too closely. Gaussians with a height of 1.2 kJ mol^{-1} were deposited every 1000 time steps, with a bias factor set to 8 at 300 K.

The free energy profiles associated with the dynamics of cation in-plane diffusion and desorption processes in K-mica systems were calculated using well-tempered metadynamics simulations. For K^+, two CVs were used: the K-Al coordination number, used to track cavity relocation across 1-Al and 2-Al ditrigonal cavity sites (CV1), and a CV designed to count the number of IS K^+ ions (CV2). Specifically, the coordination number in CV1 is defined as

$$\mathrm{CN} = \frac{1-(\frac{r-d_0}{r_0})^n}{1-(\frac{r-d_0}{r_0})^m} \tag{S1}$$

where r is the distance between the two selected species. The parameters d_0, r_0, n, and m were carefully chosen to capture only the first coordination shell, with their values listed in Table S2. For CV2, the Between function with a Gaussian kernel, as implemented in the PLUMED package, was applied between Al atoms located at the center of the mica slab and Na^+ or K^+.[13] Here, the parameters were carefully chosen to count cations located within 7 Å of the center of the slab, thus including only inner sphere (IS) species and excluding outer sphere (OS) species. In other words, CV2 counts the number of Na^+ or K^+ ions occupying IS1 and IS2 positions. The upper, lower and smear parameters used for both cations are listed in Table S2. The same metadynamics protocol was applied for Na-mica systems, but using CV2 only. For both simulations, Gaussians with a height of 1.2 kJ mol^{-1} were deposited every 1000 time steps, with a bias factor of 8 at 300 K.

Table S2: Parameters for the Enhanced Sampling CVs

Coordination Number	n	m	r_0	d_0
K–Al	6	12	0.7	3.7
Na–Al	6	12	0.7	3.3
Between	Upper	Lower	Smear	-
Cation–Al	7.0	-7.0	0.05	-

2 DP Error Analysis and Validation

2.1 Training and Validation Set Errors

The parity plots generated for the training set using one of the DPs are shown in Figures S1 and S2 for K-mica and Na-mica systems interfaced with electrolyte solutions. In general, the energy errors range between 0.7 and 1.0 meV atom^{-1}, while the force errors range between 0.13 and 0.14 eV Å^{-1}, comparable to those reported in previous work for K-mica–water interfaces.[1] The validation set was constructed by running 20 ns DPMD simulations at 300 K and sampling configurations along the trajectories. The energies and forces of these configurations were predicted using both the SCAN functional and the DPs, with the corresponding parity plots shown in Figure S3. Overall, the energy and force errors for the validation set are comparable to, or slightly smaller than, those in the training set, indicating that the DPs can accurately predict energies and forces for the relevant mica systems.

2.2 Validations on Structures

To ensure that trained DPs reproduce structures consistent with those generated using the SCAN functional, we performed both DPMD and AIMD simulations on two systems: KCl solution consisting of one KCl and 64 H_2O molecules, and a K-mica slab interfaced with 0.5 M NaCl solution. The duration of the AIMD trajectories was at least 10 ps. Radial distribution functions (RDFs) calculated from the resulting trajectories are shown in Figure S4. For the KCl solution, the trained DPs are able to reproduce essentially identical K^+ hydration structures compared to the AIMD simulations. Similarly, for the K-mica system, the RDFs obtained from both simulations show excellent agreement. These results further verify that the DPs reproduce SCAN-quality structural properties at substantially reduced computational cost.

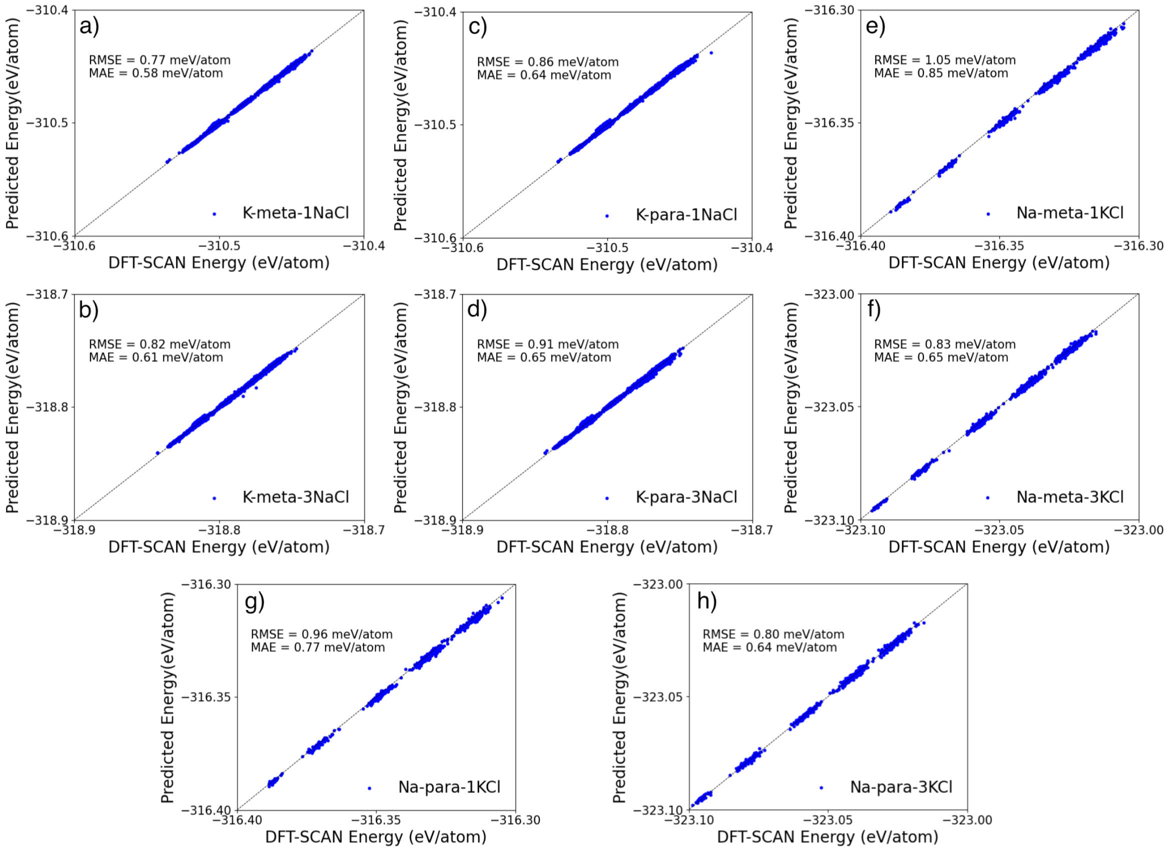

Figure S1: Parity plots comparing total energies predicted by the trained DP and SCAN for various mica systems using configurations from the **training set**. Each point represents the total energy of a single configuration, with closer agreement to the diagonal line indicating higher DP accuracy. Root mean squared errors (RMSE) and mean absolute errors (MAE) are reported in each panel.

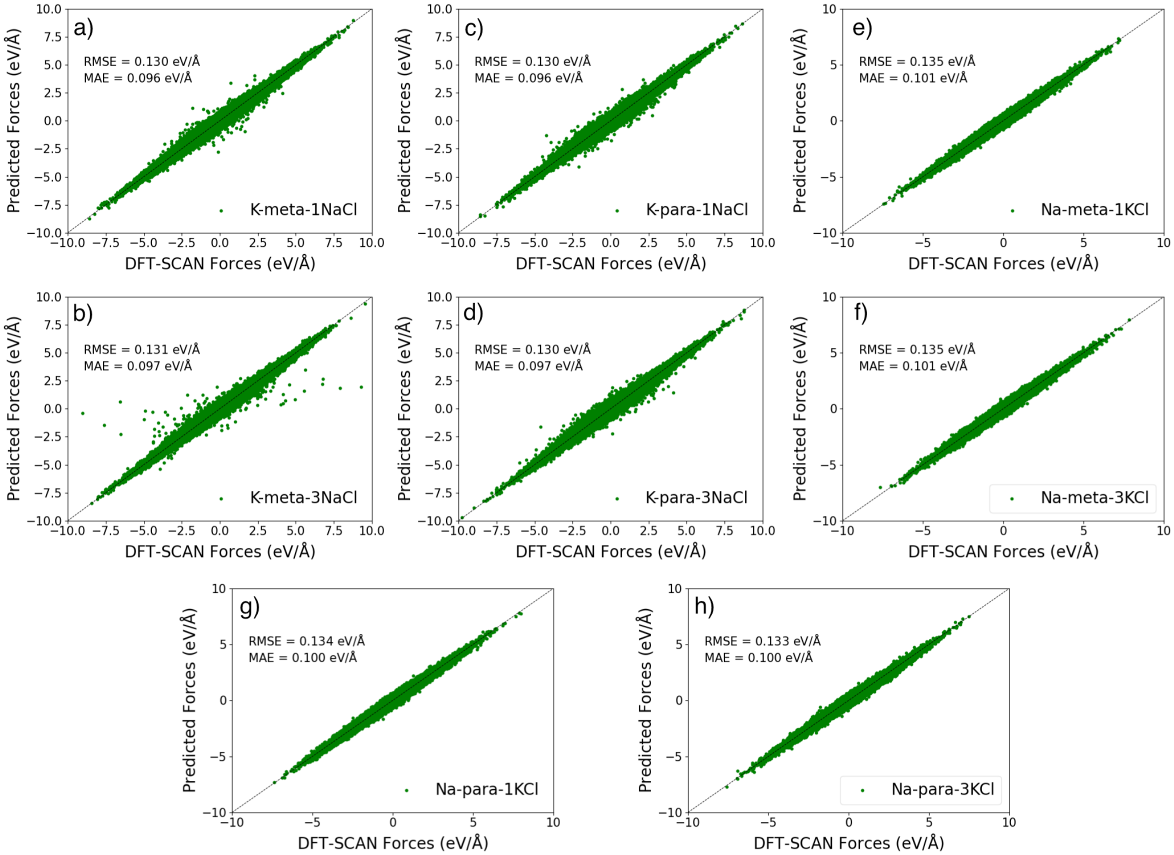

Figure S2: Parity plots comparing forces predicted by the trained DP and SCAN for various mica systems using configurations from the **training set**. Each point represents the total energy of a single configuration, with closer agreement to the diagonal line indicating higher DP accuracy. Root mean squared errors (RMSE) and mean absolute errors (MAE) are reported in each panel.

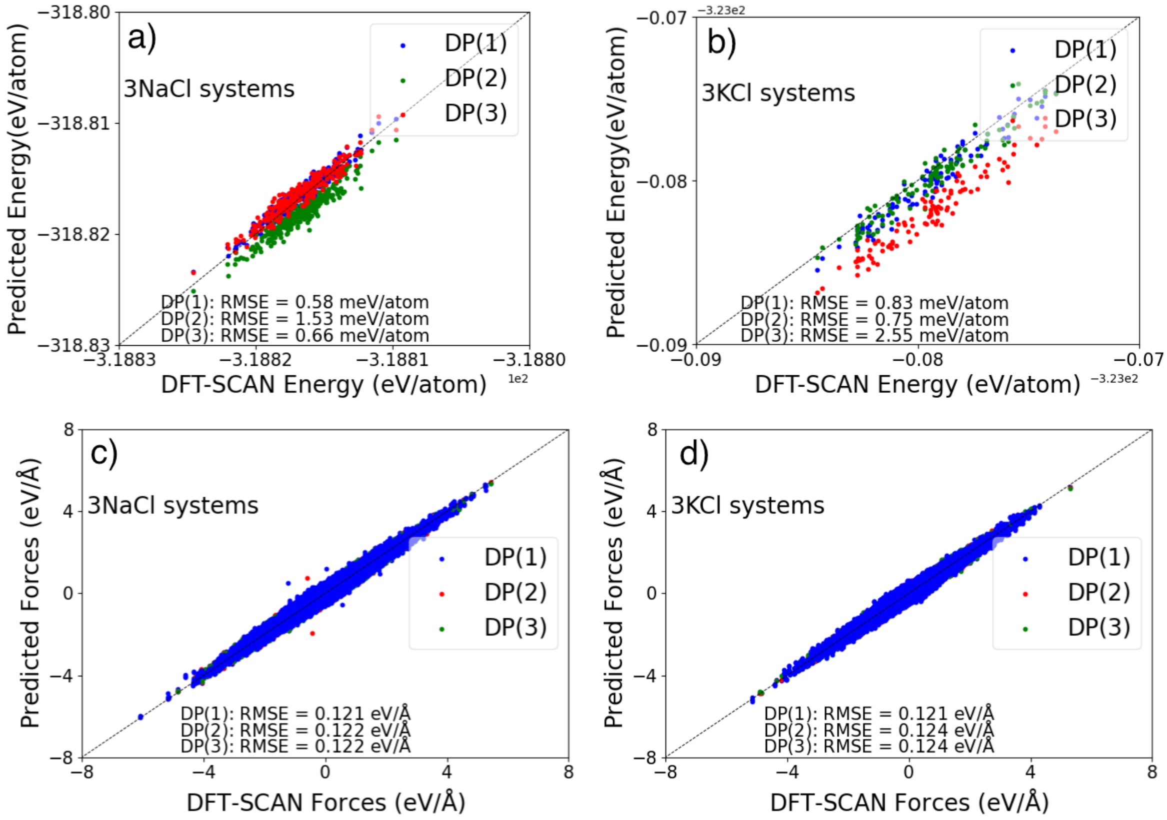

Figure S3: Parity plots of total energies (a,b) and forces (c,d) predicted by all three DPs versus the SCAN functional for configurations drawn from the **validation set**. Specifically, the validation set consists of K-mica interfaced with 3 NaCl solution and Na-mica interfaced with 3 KCl solution.

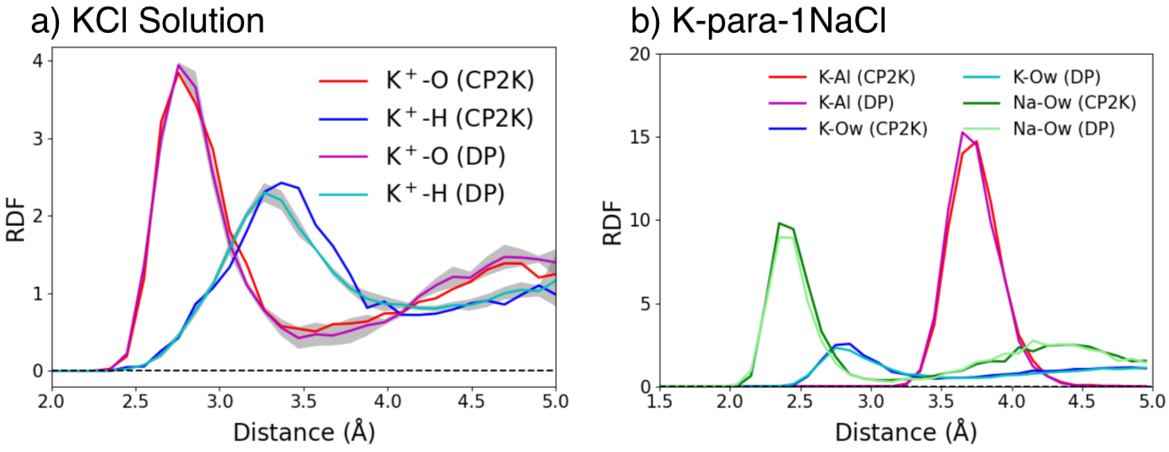

Figure S4: (a) Radial distribution functions of K–O and K–H pairs for the KCl solution system calculated using AIMD and DPMD. The shaded areas indicate the standard deviation across the three independent DPs. (b) K–Al, K–O_w, Na–O_w radial distribution functions for K-mica (para-Al) interfaced with 1 NaCl solution generated using AIMD and DPMD.

3 Additional Analysis of the Equilibrium Trajectories

The RDFs of all K-mica and Na-mica systems investigated in this work are shown in Figures S5 and S6 for the O_{surf}–O_w, Al_{surf}–O_w, Si_{surf}–O_w, K^+–O_w, and Na^+–O_w pairs. All systems exhibit similar hydration structures, with the height of the first peak in the K^+–O_w and Na^+–O_w RDFs effectively reflecting the number of cations desorbed from the surface and fully solvated by water. Interestingly, the surface Al arrangement does not induce any meaningful changes in the overall solvation structures.

Density profiles of Na^+, K^+, Cl^-, and O_w as a function of distance from the mica surface are presented in Figures S7 and S8 for K-mica and Na-mica systems, respectively. For K-mica, K^+ predominantly remains in IS1 position even when interfaced with a 3 NaCl solution slab. The added Na^+ ions occupy IS1, IS2, and OS states, consistent with their stronger preference for water solvation. We also observe that Cl^- ions typically reside between the IS2 and OS regions, providing charge screening against the accumulation of positive charge on the surface, induced by co-adsorption of multiple cations. Lastly, O_w exhibits a distinct surface-normal density profile with a pronounced double peak located around 3-4 Å. This double peak feature is largely washed out when the surface is interfaced with a 3 NaCl solution slab due to the presence of additional cations and anions.

For Na-mica, Na^+ yields a respectable OS population even when the surface is in contact with pure water. Upon introduction of 3 KCl solution, the added K^+ ions predominantly occupy the IS1 state, with Cl^- ions located either between the IS and OS regions or beyond the OS region. For water oxygen atoms, the double peak feature is absent in all Na-mica systems, confirming that such a feature is induced by a structured water layer solvating IS K^+ cations in the absence of Na^+. The first and second peaks of the O_w density decrease when the system is interfaced with 3 NaCl solution, likely due to the increased population of ions adjacent to the surface. We also note that Al arrangements fail to induce any changes in the overall density profiles of all species.

In the main text, we point out that the IS1 position corresponds to cations located at

the center of the ditrigonal cavities, whereas IS2 states are positioned at the cavity edges. Although this assignment has been established in many previous studies, we carried out additional verification by calculating the in-plane (XY) distance distributions of Na^+–Si_{surf} and Na^+–O_{surf} pairs, shown in Figure S9. The underlying rationale is that the cations located in the center of the cavity should exhibit a first peak in the XY distance distribution beyond 1 Å while the cations positioned towards the edge of the cavity should yield populations at distances close to 0 Å. Consistent with this expectation, IS1 Na^+ indeed exhibits a first peak beyond 1 Å while IS2 produces a significant population at small distances, confirming the positional trends of the IS1 and IS2 states.

We show the temporal evolution of the cation z-coordinates in both K-mica and Na-mica systems in Figures S10 and S11. These plots effectively capture cation adsorption and desorption dynamics throughout the DPMD trajectories. As discussed in the main text, the chaotropic K^+ typically prefers to remain adsorbed at the IS position over the course of the simulations, while Na^+ exhibits much more fluxional behavior. When mica slabs are interfaced with electrolyte solutions, both K^+ and Na^+ become more prone to desorption, as reflected by increased mobility, with the effect being more pronounced for Na^+.

Lastly, we calculated ion density profiles as a function of distance from the mica surface using configurations collected at earlier and later times from the 20 ns trajectories for systems at higher electrolyte concentrations, as shown in Figure S12. Specifically, profiles labeled as "Early" were obtained from data collected between 10 and 15 ns, whereas the "Late" profiles were obtained from data collected between 15 and 20 ns. The differences between the early and late profiles are small and fall within the fluctuation range observed across different DP potentials. Thus, based on these profiles, we conclude that the 20 ns simulations are sufficient to sample equilibrium configurations.

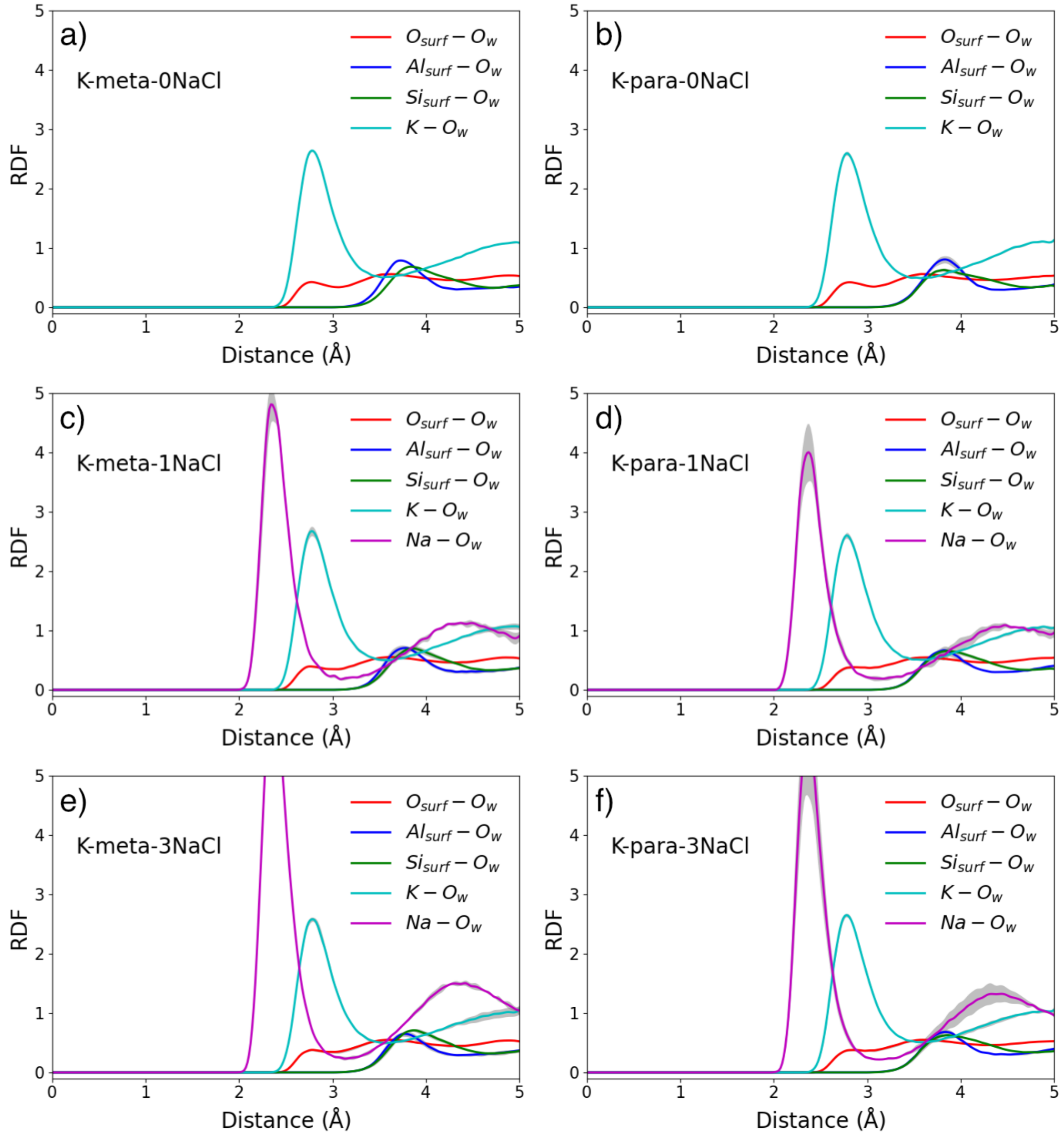

Figure S5: Radial distribution functions of O_{surf}–O_w, Al_{surf}–O_w, Si_{surf}–O_w, K^+–O_w, and Na^+–O_w pairs for K-mica interfaced with solutions containing 0, 1, and 3 NaCl pairs. Shaded regions indicate the standard deviation across the three independent DPs. System naming follows the convention (Na- or K-mica)-(meta or para Al arrangement)-(0, 1, or 3 NaCl or KCl solution).

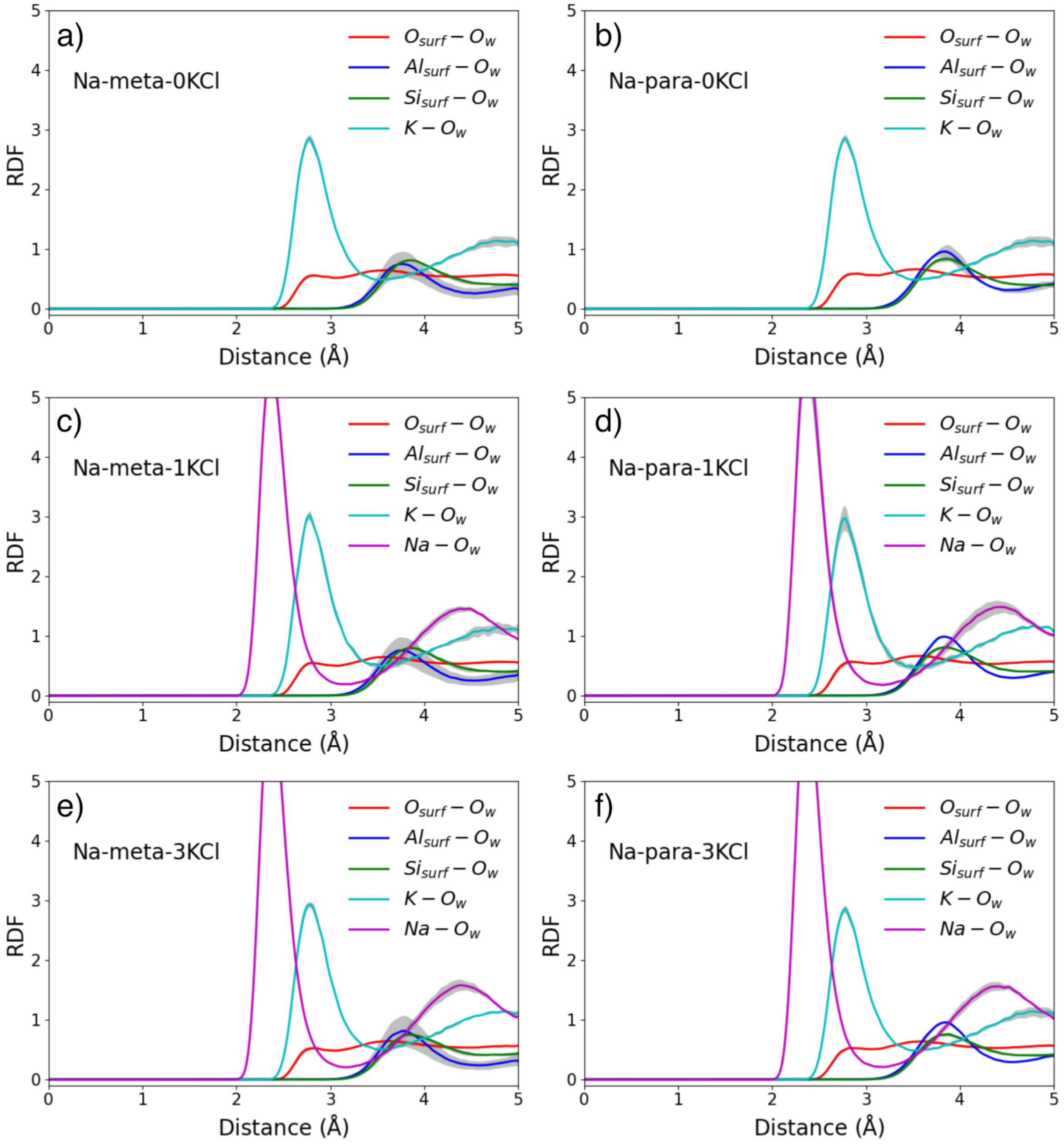

Figure S6: Radial distribution functions of O_{surf}–O_w, Al_{surf}–O_w, Si_{surf}–O_w, K^+–O_w, and Na^+–O_w pairs for Na-mica interfaced with solutions containing 0, 1, and 3 KCl pairs. Shaded regions indicate the standard deviation across the three independent DPs. System naming follows the convention (Na- or K-mica)-(meta or para Al arrangement)-(0, 1, or 3 NaCl or KCl solution).

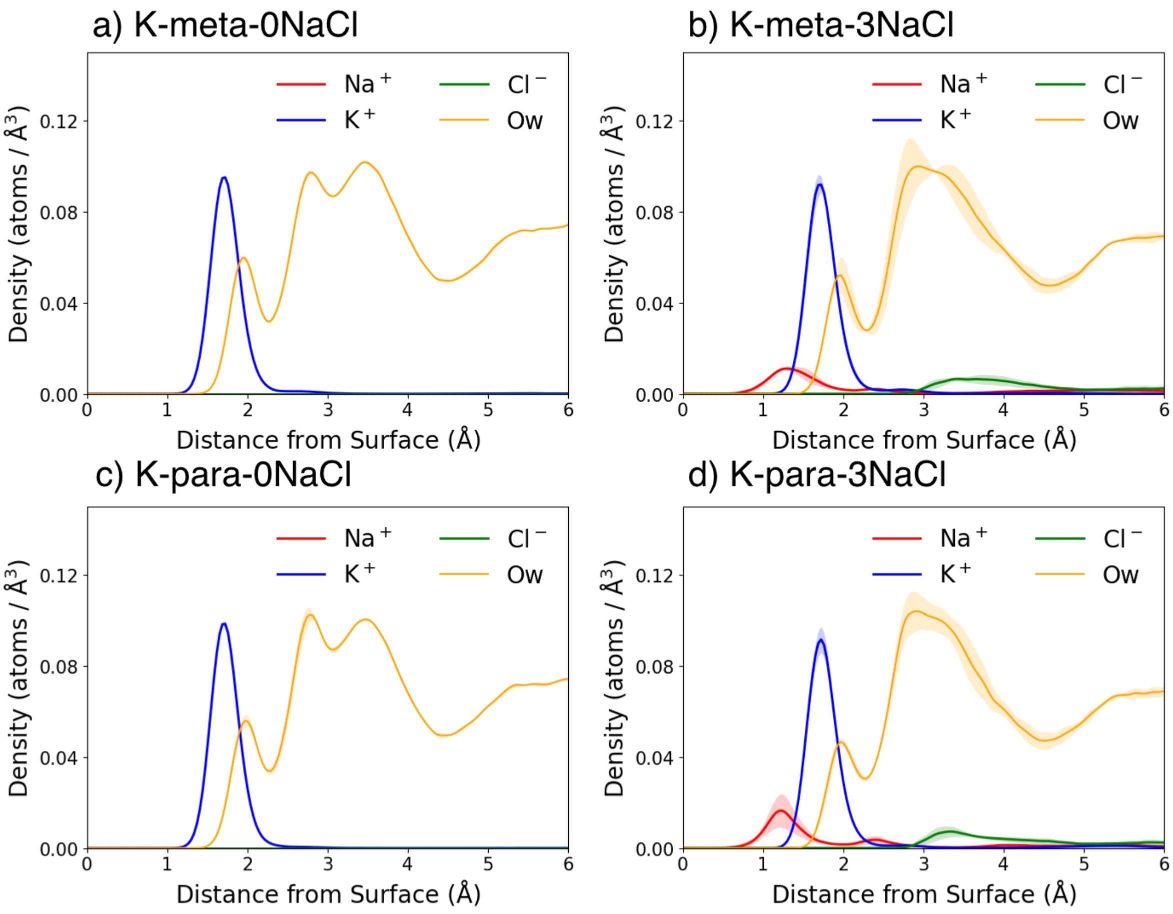

Figure S7: Density profiles of K^+, Na^+, Cl^-, and O_w as a function of distance from the mica surface for K-mica slabs interfaced with either pure water or solution containing 3 NaCl pairs. Shaded regions indicate the standard deviation across the three independent DPs.

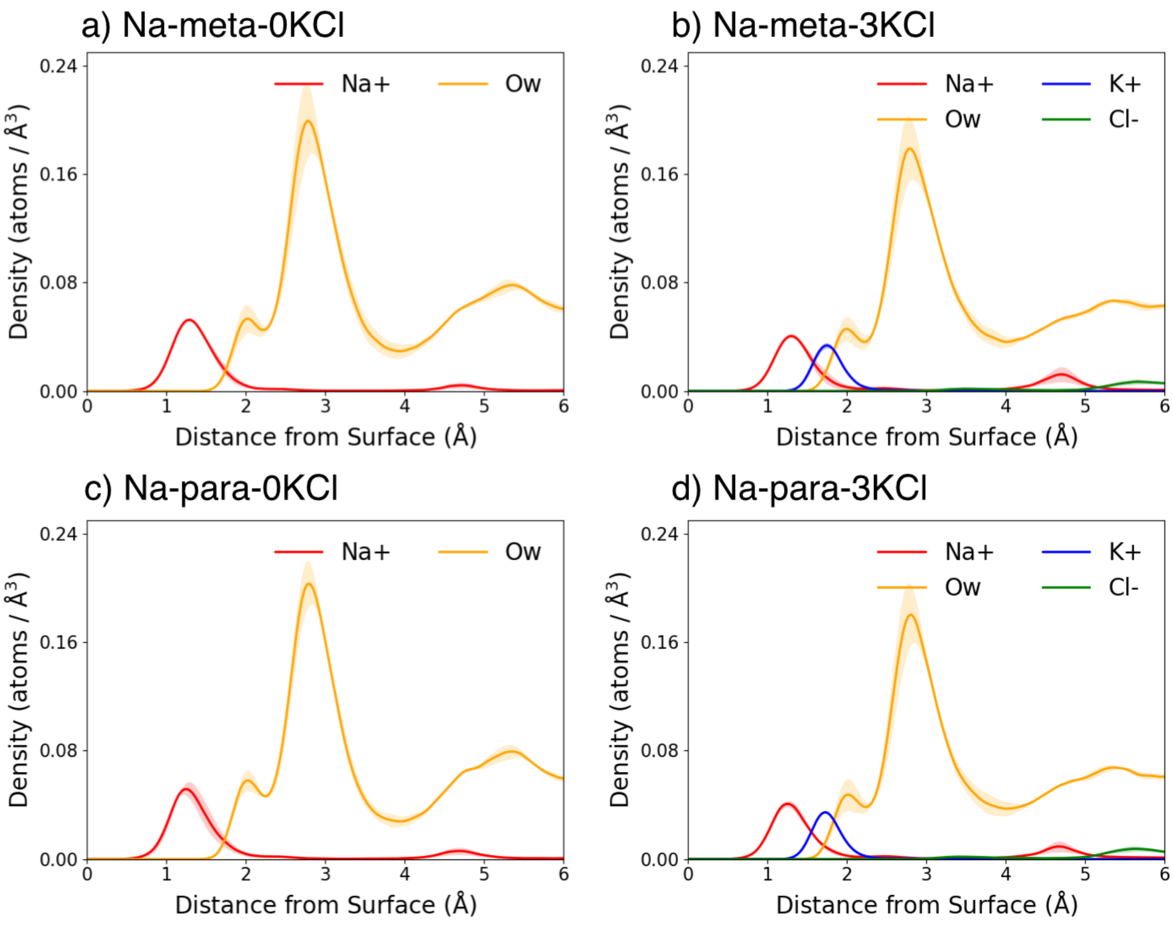

Figure S8: Density profiles of K^+, Na^+, Cl^-, and O_w as a function of distance from the mica surface for Na-mica slabs interfaced with either pure water or solution containing 3 KCl pairs. Shaded regions indicate the standard deviation across the three independent DPs.

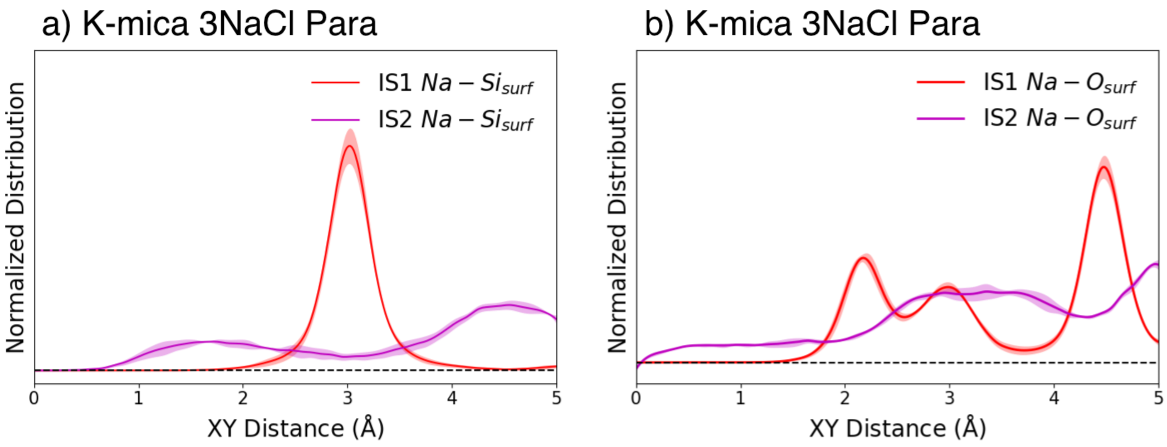

Figure S9: In-plane (XY) distance distributions of the Na–Si_{surf} pair for Na^+ ions located at either the IS1 and IS2 positions. Shaded regions indicate the standard deviation across the three independent DPs.

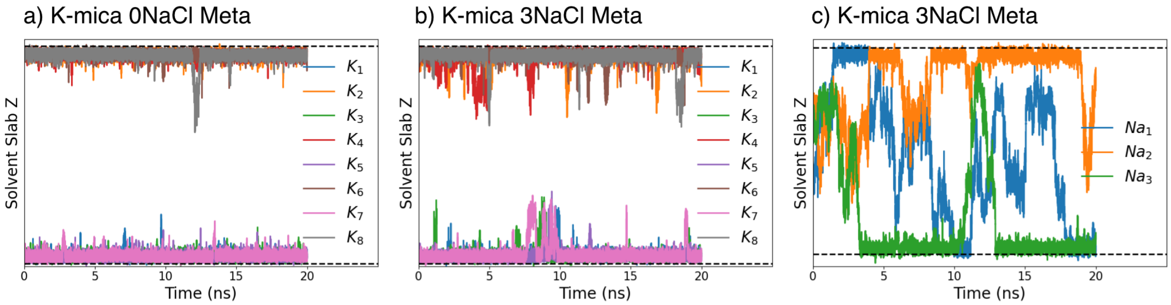

Figure S10: z-axis positional dynamics of all K^+ and Na^+ ions in K-mica systems with the meta-Al arrangement. The two horizontal dashed lines near the top and bottom of each panel represent the two surfaces of the mica slab.

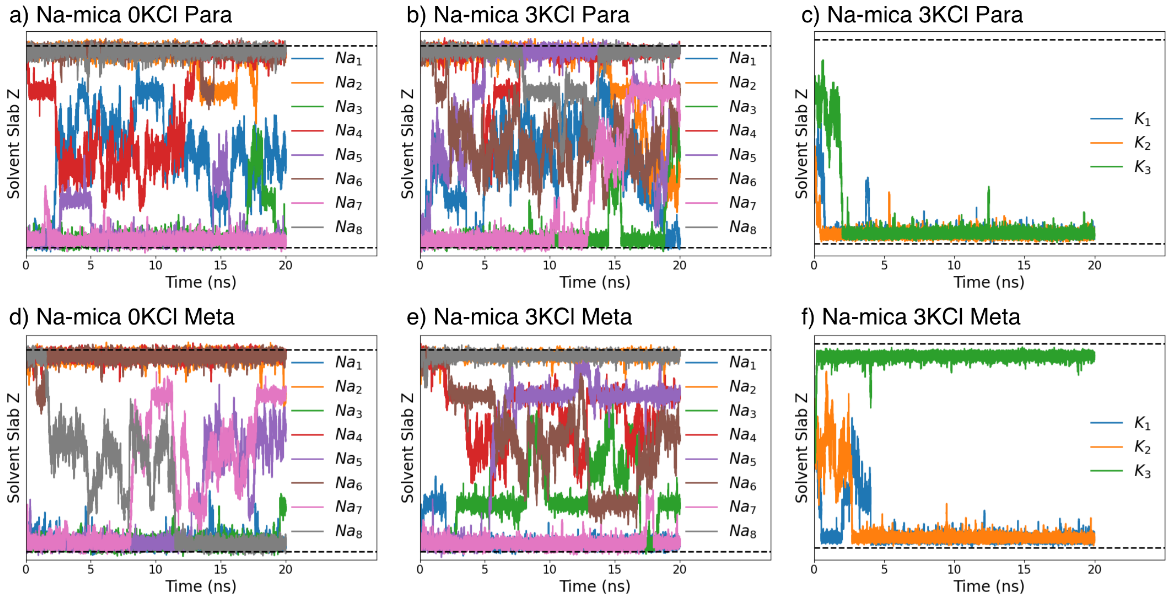

Figure S11: z-axis positional dynamics of all K^+ and Na^+ ions in Na-mica systems with both the meta and para-Al arrangements. The two horizontal dashed lines near the top and bottom of each panel represent the two surfaces of the mica slab.

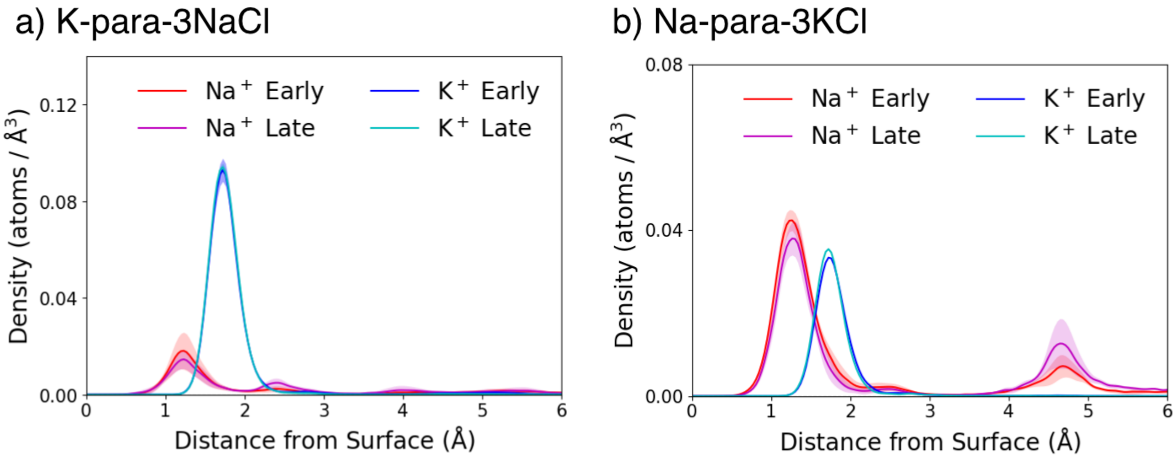

Figure S12: Density profiles of K^+ and Na^+ as a function of distance from the mica surface. The early profiles were calculated from data collected between 10 and 15 ns, and the late profiles were calculated from data collected between 15 and 20 ns.

4 Additional Analysis of the Enhanced Sampling Simulations

For most of the results presented in both the main text and the SI, the Al arrangement appears to have minimal impact on the majority of the observed properties. To further examine this potential effect, we first compared the surface normal Na^+ density profiles for K-mica interfaced with 1 NaCl solution, shown in Figures S13a and S13b. Interestingly, the meta-Al exhibits a more pronounced IS2 and OS population compared to the para-Al. To substantiate this trend, we calculated the free energy profiles for Na^+ desorption, presented in Figure S13c. Here, both the IS2 and OS states are lower in free energy for the meta arrangement, providing further support to this observation. As discussed in the previous section, the transition from IS1 to IS2 states corresponds to cations moving from the center of the ditrigonal cavities toward the edge of the cavity. This process is facilitated when Al atoms are more spatially clustered, as in the meta-Al shown in Figure S13d. In contrast, the more symmetric Al placement in the para arrangement tends to stabilize the IS1 position.

To estimate the free energy associated with K^+–Na^+ exchange in K-mica systems, we performed well-tempered metadynamics simulations using the two CVs introduced in the Methods section, $z_1 + z_2$ and $z_1 - z_2$, which represent adsorbed and desorbed states of the selected Na^+ and K^+ ions. The resulting two dimensional free energy profiles are shown in Figures S14a and S14b for K-mica interfaced with a 1 NaCl solution slab for both Al arrangements. The leftmost basin with the lowest free energy corresponds to a state in which both cations are adsorbed in the IS state on the surface, while movement toward upper right or lower right regions corresponds to K^+ or Na^+ desorption, respectively. One-dimensional projections of these free energy profiles are shown in Figures S14c and S14d. Regardless of the Al arrangements, Na^+ desorption is more favorable than K^+ desorption, and the resulting K^+–Na^+ exchange free energy is approximately 10 kJ mol^{-1}, in good agreement with experimental values.

Next, we present full two-dimensional free energy profiles from well-tempered metadynamics simulations that sample cation in-plane cavity diffusion and surface normal speciation dynamics, with the results for K-mica systems shown in Figures S15 and S16 for both Al arrangements. As discussed in the main text, K^+ favors full IS adsorption while exhibiting frequent cavity relocation between 1-Al and 2-Al sites, thereby occupying both 1-Al and 2-Al ditrigonal cavities on average. Upon introduction of 3 NaCl solution, K^+ migration toward the OS and desorbed states becomes more favorable, accompanied by a subtle increase in occupation of 1-Al cavities. The added Na^+ ions are much more prone to desorption, as states with one or two adsorbed Na^+ are preferred over the fully adsorbed state. Similar to K^+, Na^+ also exhibits mixed occupation of 1-Al and 2-Al cavities, indicating active cavity relocation dynamics. All of these observations are in good agreement with the interpretation presented in the main text.

For Na-mica systems, we instead employed a single CV that tracks the number of IS adsorbed K^+ or Na^+ ions, and the resulting free energy profiles are shown in Figure S17. Here, Na^+ escapes from the IS state much more readily, with the most favorable states corresponding to configurations in which 4-6 Na^+ remain adsorbed in the IS state. Introducing 3 KCl electrolyte solution further enhances Na^+ desorption, stabilizing states with even fewer IS Na^+ ions. In contrast, K^+ prefers full IS adsorption even in Na-mica systems. Additionally, no significant differences are observed between the meta-Al and para-Al arrangements.

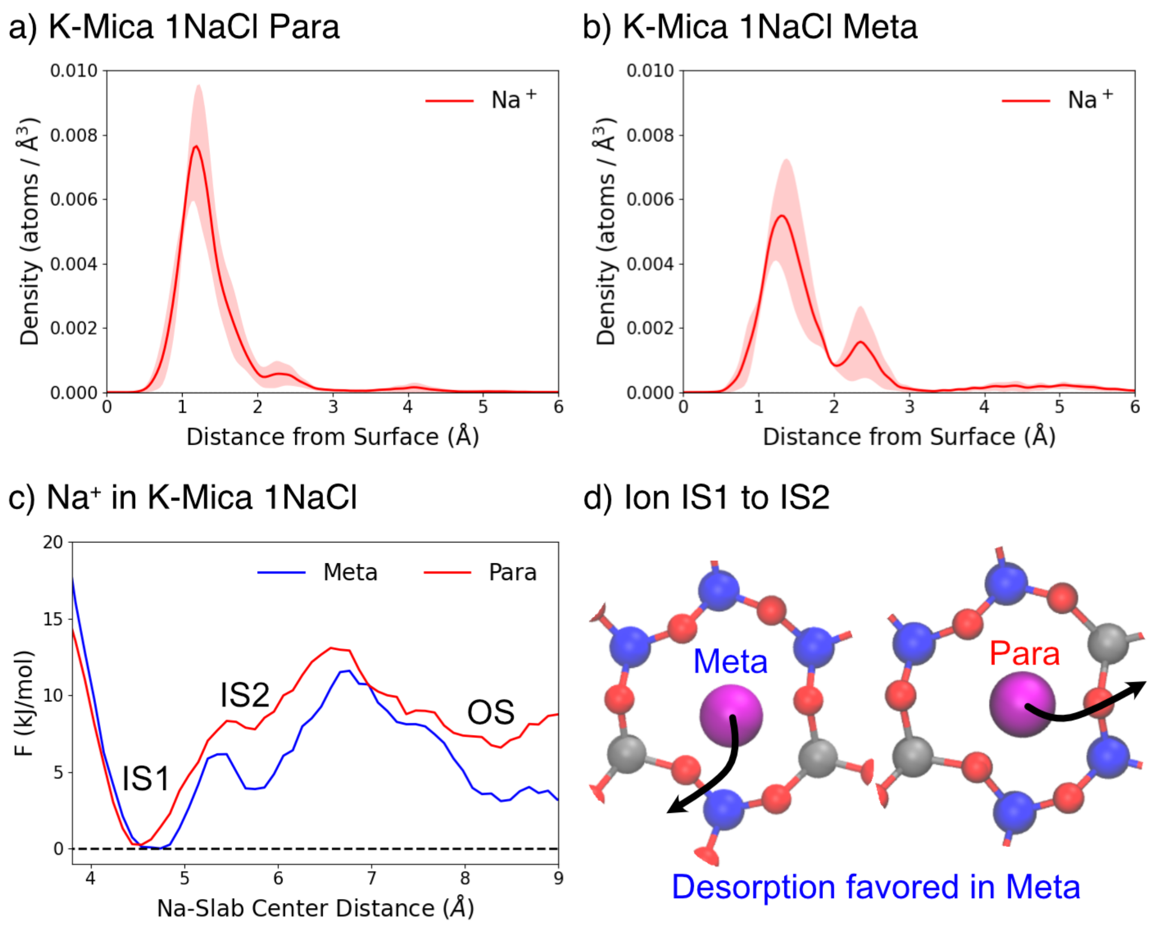

Figure S13: (a,b) Na^+ density profiles as a function of distance from the surface for K-mica slabs with meta-Al and para-Al arrangements interfaced with a 1 NaCl solution slab. Shaded regions indicate the standard deviation across the three independent DPs. (c) Free energy profiles of Na^+ desorption for the two Al arrangements of the same system. These profiles demonstrate relative free energy differences between distinct adsorption states, including IS1, IS2, and OS. (d) Schematic illustration of Na^+ migration from the IS1 to IS2 position for both meta-Al and para-Al arrangements.

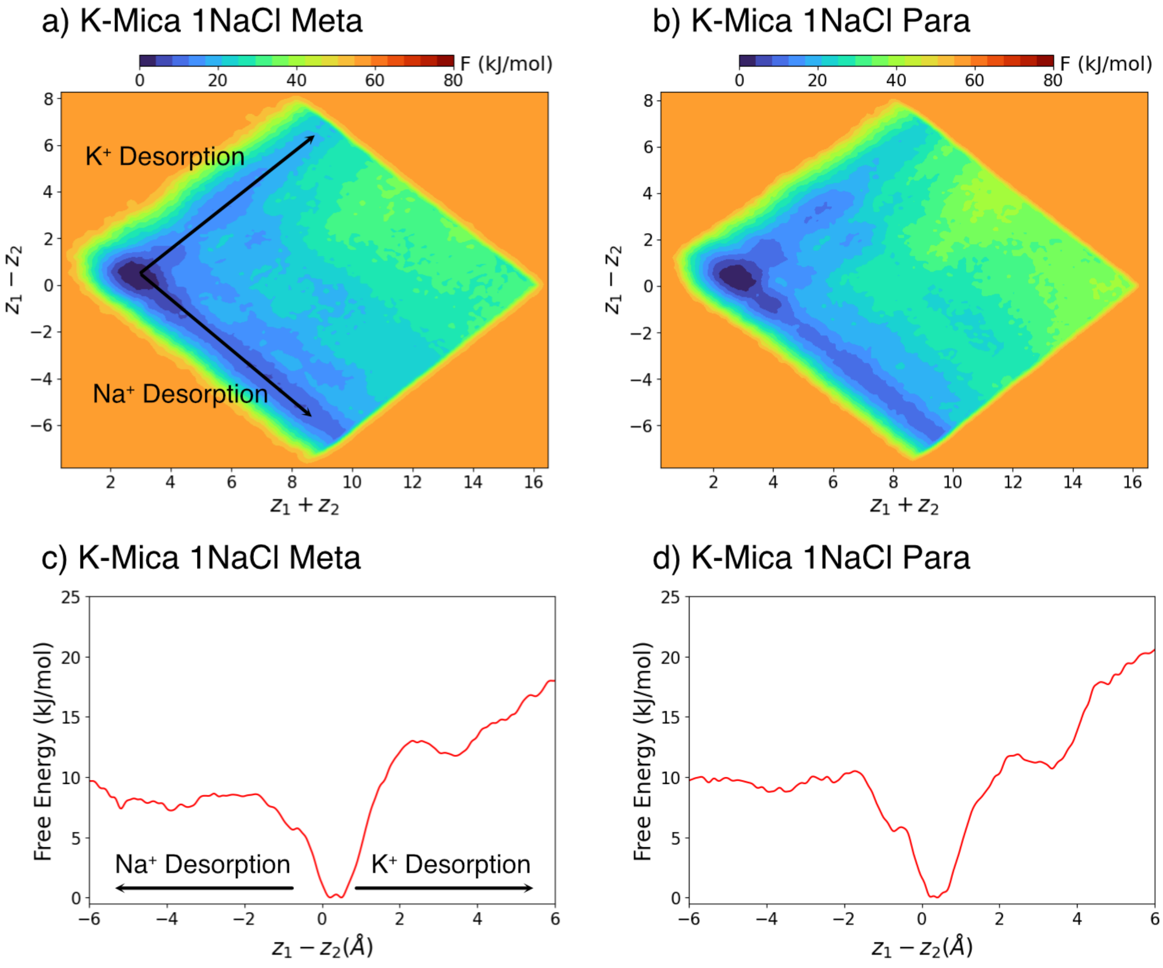

Figure S14: (a,b) Two dimensional free energy profiles along the two CVs describing adsorption and desorption of selected Na^+ and K^+ ions for meta and para K-mica slabs interfaced with 1 NaCl solution. Darker blue regions correspond to lower free energy, while redder hues indicate higher free energy. (c,d) One dimensional projection of the full free energy profiles onto one of the CVs. For all free energy profiles, the directions of Na^+ and K^+ desorption are represented with black arrows.

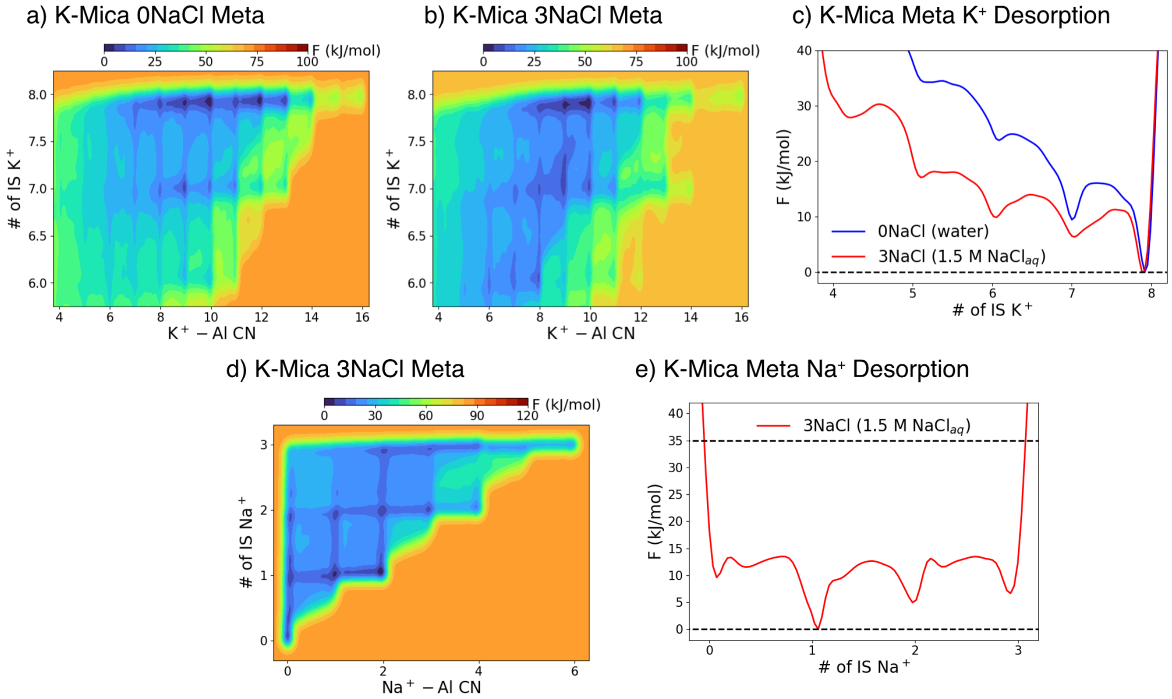

Figure S15: (a,b) Two dimensional free energy profiles along two CVs describing K^+ IS and cavity diffusion dynamics for K-mica systems with the meta-Al arrangement. Darker blue regions correspond to lower free energy, while redder hues indicate higher free energy. (c) One-dimensional projection of the free energy surface onto the CV representing the number of IS adsorbed K^+ ions. (d) Two dimensional free energy profiles of Na^+ IS and cavity relocation dynamics for the same system. (e) One dimensional projection of the free energy surface onto the CV representing the number of IS adsorbed Na^+

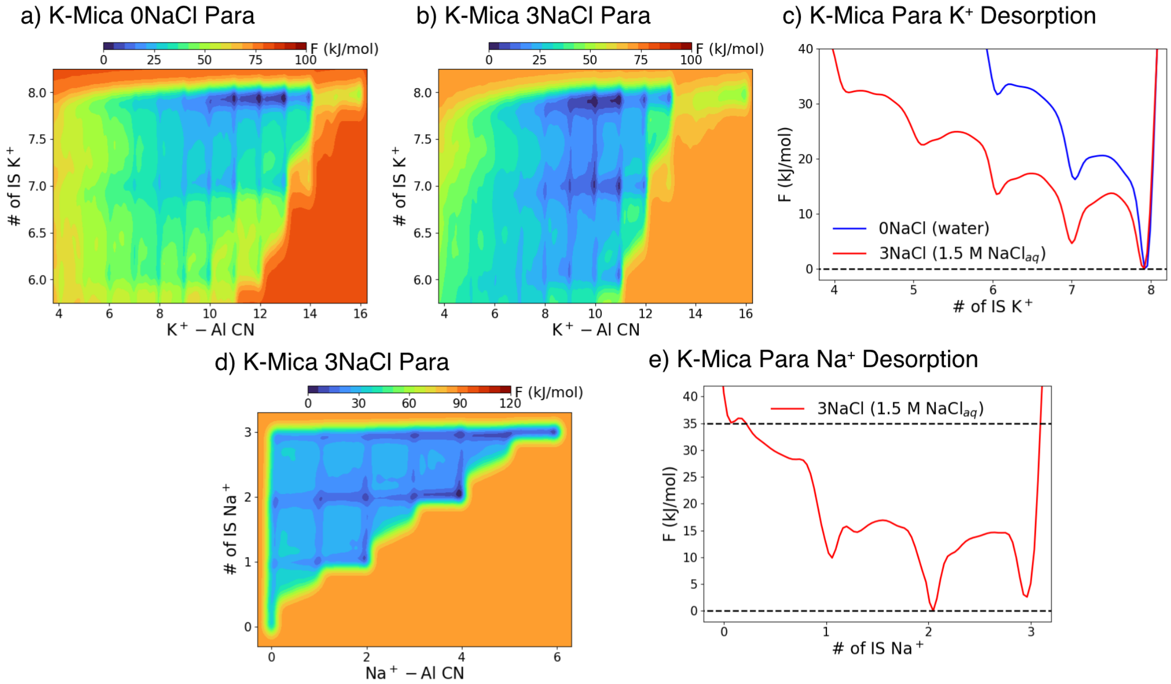

Figure S16: (a,b) Two dimensional free energy profiles along two CVs describing K^+ IS and cavity diffusion dynamics for K-mica systems with the para-Al arrangement. Darker blue regions correspond to lower free energy, while redder hues indicate higher free energy. (c) One-dimensional projection of the free energy surface onto the CV representing the number of IS adsorbed K^+ ions. (d) Two dimensional free energy profiles of Na^+ IS and cavity relocation dynamics for the same system. (e) One dimensional projection of the free energy surface onto the CV representing the number of IS adsorbed Na^+

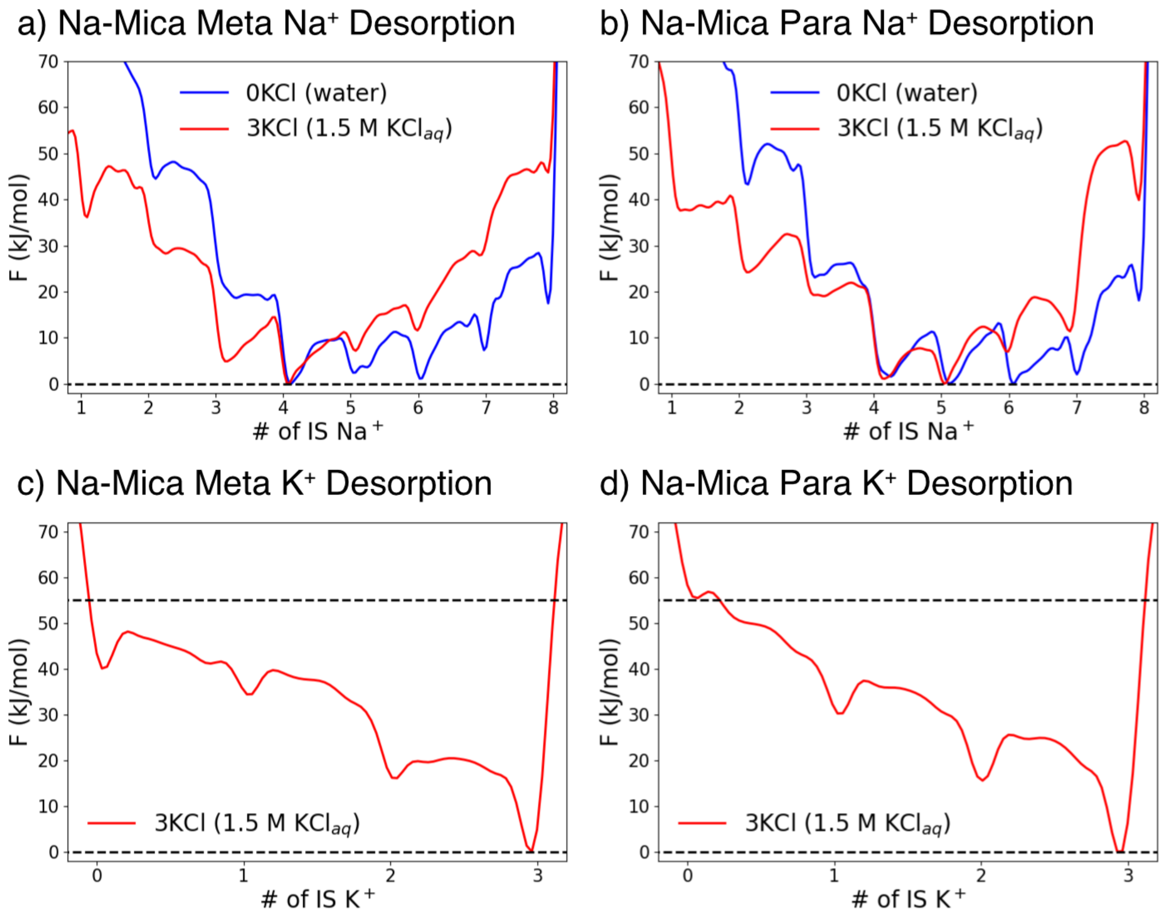

Figure S17: (a,b) Free-energy profiles of sequential Na^+ migration from IS adsorption for the meta-Al and para-Al Na-mica slabs interfaced with either 0 KCl (blue curves) or 3 KCl (red curves) solutions. (c,d) Free-energy profiles of sequential K^+ migration for the same systems.

References

(1) Raman, A. S.; Selloni, A. Insights into the structure and dynamics of K+ ions at the muscovite–water interface from machine learning potential simulations. J. Chem. Phys. **2024**, 160.

(2) Raman, A. S.; Selloni, A. An ab-initio deep neural network potential for accurate large-scale simulations of the muscovite mica-water interface. Molecular Phys. **2025**, 123, e2365430.

(3) Sun, J.; Ruzsinszky, A.; Perdew, J. P. Strongly constrained and appropriately normed semilocal density functional. Phys. Rev. Lett. **2015**, 115, 036402.

(4) Giannozzi, P.; Baroni, S.; Bonini, N.; Calandra, M.; Car, R.; Cavazzoni, C.; Ceresoli, D.; Chiarotti, G. L.; Cococcioni, M.; Dabo, I. et al. QUANTUM ESPRESSO: a modular and open-source software project for quantumsimulations of materials. J. Physics: Condensed Matter **2009**, 21, 395502.

(5) Hamann, D. Optimized norm-conserving Vanderbilt pseudopotentials. Phys. Rev. B—condensed Matter Mater. Phys. **2013**, 88, 085117.

(6) Kühne, T. D.; Iannuzzi, M.; Del Ben, M.; Rybkin, V. V.; Seewald, P.; Stein, F.; Laino, T.; Khaliullin, R. Z.; Schütt, O.; Schiffmann, F. et al. CP2K: An electronic structure and molecular dynamics software package-Quickstep: Efficient and accurate electronic structure calculations. J. Chem. Phys. **2020**, 152.

(7) Grimme, S.; Antony, J.; Ehrlich, S.; Krieg, H. A consistent and accurate ab initio parametrization of density functional dispersion correction (DFT-D) for the 94 elements H-Pu. J. Chem. Phys. **2010**, 132.

(8) Goedecker, S.; Teter, M.; Hutter, J. Separable dual-space Gaussian pseudopotentials. Phys. Rev. B **1996**, 54, 1703.

(9) Martyna, G. J.; Klein, M. L.; Tuckerman, M. Nosé–Hoover chains: The canonical ensemble via continuous dynamics. J. Chem. Phys. **1992**, 97, 2635–2643.

(10) Zhang, L.; Lin, D.-Y.; Wang, H.; Car, R.; E, W. Active learning of uniformly accurate interatomic potentials for materials simulation. Phys. Rev. Mater. **2019**, 3, 023804.

(11) Zhang, L.; Han, J.; Wang, H.; Car, R.; E, W. Deep potential molecular dynamics: a scalable model with the accuracy of quantum mechanics. Phys. Rev. Lett. **2018**, 120, 143001.

(12) Zhang, C.; Calegari Andrade, M. F.; Goldsmith, Z. K.; Raman, A. S.; Li, Y.; Piaggi, P. M.; Wu, X.; Car, R.; Selloni, A. Molecular-scale insights into the electrical double layer at oxide-electrolyte interfaces. Nat. Commun. **2024**, 15, 10270.

(13) Bonomi, M.; Branduardi, D.; Bussi, G.; Camilloni, C.; Provasi, D.; Raiteri, P.; Donadio, D.; Marinelli, F.; Pietrucci, F.; Broglia, R. A. et al. PLUMED: A portable plugin for free-energy calculations with molecular dynamics. Computer Phys. Commun. **2009**, 180, 1961–1972.
